# The Development and Atomic Structure of Zinc Oxide Crystals Grown within Polymers from Vapor Phase Precursors

**DOI:** 10.1021/acsnano.4c02846

**Published:** 2024-07-03

**Authors:** Inbal Weisbord, Maya Barzilay, Ruoke Cai, Edmund Welter, Alexei Kuzmin, Andris Anspoks, Tamar Segal-Peretz

**Affiliations:** † Department of Chemical Engineering, Technion − Israel Institute of Technology, 3200003 Haifa, Israel; ‡ Deutsches Elektronen-Synchrotron − A Research Centre of the Helmholtz Association, Notkestrasse 85, D-22607 Hamburg, Germany; § Institute of Solid State Physics, University of Latvia, Kengaraga Street 8, LV-1063 Riga, Latvia

**Keywords:** Sequential Infiltration Synthesis, Vapor Phase Infiltration, Scanning Transmission Electron Microscopy, X-ray Absorption Near Edge Structure, Hybrid Organic−Inorganic, Polymers

## Abstract

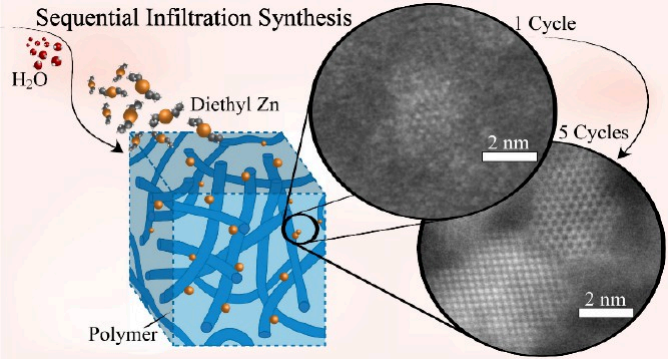

Sequential infiltration synthesis (SIS), also known as vapor phase infiltration (VPI), is a quickly expanding technique that allows growth of inorganic materials within polymers from vapor phase precursors. With an increasing materials library, which encompasses numerous organometallic precursors and polymer chemistries, and an expanding application space, the importance of understanding the mechanisms that govern SIS growth is ever increasing. In this work, we studied the growth of polycrystalline ZnO clusters and particles in three representative polymers: poly(methyl methacrylate), SU-8, and polymethacrolein using vapor phase diethyl zinc and water. Utilizing two atomic resolution methods, high-resolution scanning transmission electron microscopy and synchrotron X-ray absorption spectroscopy, we probed the evolution of ZnO nanocrystals size and crystallinity level inside the polymers with advancing cycles—from early nucleation and growth after a single cycle, through the formation of nanometric particles within the films, and to the coalescence of the particles upon polymer removal and thermal treatment. Through *in situ* Fourier transform infrared spectroscopy and microgravimetry, we highlight the important role of water molecules throughout the process and the polymers’ hygroscopic level that leads to the observed differences in growth patterns between the polymers, in terms of particle size, dispersity, and the evolution of crystalline order. These insights expand our understanding of crystalline materials growth within polymers and enable rational design of hybrid materials and polymer-templated inorganic nanostructures.

## Introduction

Sequential infiltration synthesis (SIS) is a rapidly growing method that allows growth of inorganic materials inside polymers using vapor precursors.^1−4^ Also referred to as vapor phase infiltration (VPI) and atomic layer infiltration (ALI), it is based on atomic layer deposition (ALD) chemistry and utilizes the cyclic exposure of precursors for selective and controlled reaction with polymer functional groups. SIS provides an alternative to common nanofabrication techniques as it enables a controlled, step-by-step conversion of organic nanostructures into hybrid and inorganic nanostructures. Over the past 15 years, the SIS materials library has been greatly expanded to include precursors for growth of metal oxides like AlO_*x*_,^5−15^ TiO_2_,^16−20^ ZnO,^5,14,21−28^ In_2_O_3_^29^ and more,^29−33^ and even metals like W,^5,34^ and Ru.^35^ The scope of polymers has been greatly broadened over this time period, as well, and SIS has been used to alter the inherent properties of both natural^16,36^ and synthetic polymers,^21,37−40^ including block copolymers (BCPs)^41^ and block terpolymers.^42^ Demonstrated applications included improved water purification,^10,43^ controlled mechanical^16,17,36,40,44−46^ and electrical properties,^23,30,38,39,47^ templating for nanofabrication,^11,18,48^ and imaging contrast enhancement.^9,49,50^

Mechanistic studies are imperative for an improved understanding of the SIS process and its better integration into real-world systems. Currently, the great majority of such studies were dedicated to trimethyl aluminum (TMA),^7,8,12,13,15,29^ which has been probed by microgravimetry, infrared (IR) spectroscopy, ellipsometry, electron microscopy (EM), time-of-flight-secondary ion mass spectrometry (ToF-SIMS), X-ray, and other methods, with models developed to predict and understand diffusion and growth as a function of process parameters.^12,15,51^ Recent work on In_2_O_3_ SIS has expanded the scope of mechanistic studies, probing trimethyl In (TMIn) diffusion rates compared to TMA using ellipsometry and IR spectroscopy,^29^ and probing the resultant atomic structure using TEM and various advanced X-ray measurements.^52^

ZnO was among the first materials introduced through SIS. It represents a distinct class of materials in the SIS library. While most metal oxides and metals grown to date via SIS are amorphous, ZnO is known to grow as a polycrystalline material even at room temperature.^53^ Interest in controlling its growth is owed to its piezoelectricity, its wide band gap, biocompatibility, and more.^54−56^ For those reasons, along with the high availability of the diethylzinc (DEZ) precursor in ALD systems, it was quickly implemented in a large variety of polymer-precursor pairings. It was demonstrated by Lee et al.,^16^ in their seminal paper that showed how metal oxide growth inside spider silk can increase the mechanical durability of fibers. Peng et al. showed that a poor pattern transfer is seen when growing ZnO in the poly(methyl methacrylate) (PMMA) block of polystyrene-*block*-PMMA (PS-*b*-PMMA), and how the growth selectivity can be greatly enhanced with a single cycle of AlO_*x*_ SIS.^5^ Moshonov and Frey showed good ZnO growth in poly(3-hexylthiphene) (P3HT), but also high growth and excellent selectivity in the poly(ethylene oxide) (PEO) block of lamella-forming P3HT-*b*-PEO,^22^ and later demonstrated ZnO/P3HT potential in photovoltaic systems.^21^ Nam et al. studied DEZ growth in lithography developed SU-8 templates, looking into their crystal structure and electrical properties after polymer removal.^23^

Despite the wide application space of ZnO, mechanistic studies of ZnO growth through SIS are less common. The most notable work to date focused on the ZnO growth mechanism within SU-8 and its relation to residual solvent in the polymer film.^25^ Using IR and microgravimetry, Ye et al. showed how the presence of cyclopentanone residues, the standard SU-8 solvent, results in greatly increased ZnO growth compared to γ-butyrolactone (GBL). Other work has shown how microdose infiltration synthesis (MDIS) can increase ZnO infiltration depth in PS-*b*-poly(2-vinylpyridine) (PS-*b*-P2VP), compared to a traditional infiltration protocol, and allow for accurate pattern transfer from BCPs.^39^ Thus, understanding the growth of ZnO within polymers can widen our knowledge on crystalline materials growth in SIS.

This work expands the mechanistic understanding of ZnO_*x*_ crystal development in polymers, by examining the early stage of nucleation and growth of ZnO_*x*_ within various polymer chemistries, as well as the ZnO_*x*_ crystals’ evolution with an increasing number of SIS cycles. To this end, we combined cutting-edge atomic scale characterization methods with *in situ* measurements that unravel how ZnO_*x*_ nucleates and grows inside each polymer. High-resolution scanning transmission electron microscopy (HR-STEM) enabled us to probe the growth, from the early formation of clusters to the development of polycrystalline particles, as well as their size and distribution with the polymers, revealing the onset of crystallinity in ZnO_*x*_ SIS processes. X-ray absorption near edge structure (XANES) analysis of X-ray absorption spectroscopy (XAS) enabled us to probe the coordination environment of the developing ZnO_*x*_ crystals and showed excellent agreement with the HR-STEM data. In addition, *in situ* microgravimetry measurements and *in situ* Fourier-transform infrared (FTIR) spectroscopy revealed that the majority of the growth is associated with the water content within the polymer, where the ZnO_*x*_ initial growth is related to the level of polymer hygroscopy while the later stages are dependent on the remaining water within the hybrid ZnO_*x*_-polymer film. We envision that this work will advance the fundamental understanding of crystalline materials grown from vapor phase within polymers and would enable rational, knowledge-based design of SIS processes for hybrid and inorganic nanostructures.

## Results and Discussion

**Figure 1:**
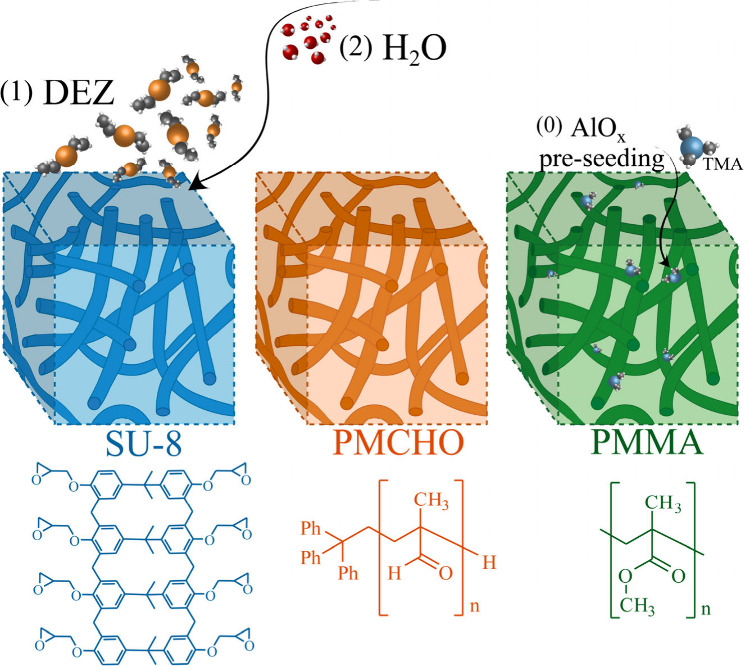
ZnO growth in different polymers illustration, showing (1) DEZ infiltration into SU-8, PMCHO and PMMA films, followed by (2) water infiltration. In PMMA films ZnO_*x*_ growth was preceded by an AlO_*x*_-seeding TMA/H_2_O cycle (0), to seed ZnO_*x*_ growth.

In order to expand the known polymer-precursor pairings, as well as gain deeper understanding of the growth of crystalline materials with SIS, we probed the interaction of the organometallic precursor diethyl Zn (DEZ) with three polymeric systems (Figure 1) using complementary methods: XAS and HR-STEM. Each of the three polymers used in this study, *i.e.*, SU-8, PMMA, and polymethacryl aldehyde (or polymethacrolein, PMCHO) was chosen for different reasons. ZnO_*x*_ SIS has already been shown through DEZ/H_2_O SIS in SU-8, an epoxy frequently used as a photoresist for nanopatterning. Growth using DEZ has also been shown in PMMA, to date the most studied polymer in the SIS field. In PMMA, however, DEZ interactions are such that little to no growth is seen, and a preliminary step of AlO_*x*_ seeding, typically with trimethyl Al (TMA), is necessary to attain significant growth.^5^ For that reason, we performed AlO_*x*_ preseeding using a single cycle of TMA/H_2_O in this study to facilitate ZnO_*x*_ growth in PMMA. The third polymer, PMCHO, was chosen due to its chemical similarity to PMMA. However, instead of the ester group in PMMA, PMCHO’s reactive group is an aldehyde, predicted to be more reactive, and therefore more likely to react with DEZ without a seeding step.

To fully understand the growth evolution with SIS cycles, we characterized the hybrid polymer-ZnO_*x*_ films after a single, three, and five SIS cycles, performed at 120 °C to increase the precursors diffusion into the films. We also hypothesized that an elevated temperature and prolonged exposure times would encourage a more homogeneous growth throughout the film depth.^15^ Each cycle consisted of a 900 s exposure to DEZ (Figure 1, step 1) in a static mode, during which time the chamber was completely sealed. Exposure to DEZ was followed by a 1200 s N_2_ purge at 20 sccm, to remove unreacted precursor. Finally, the exposure and purge steps were repeated with the coreactant, H_2_O, to form ZnO_*x*_ (Figure 1, step 2). As mentioned above, in PMMA, DEZ cycles were preceded by a single TMA/H_2_O cycle to encourage ZnO_*x*_ growth (Figure 1, step 0). Henceforth, except when crucial to the discussion, the TMA cycle will not be mentioned when describing growth in PMMA, simply stating subsequent DEZ cycles. Each system was also characterized after 5 SIS cycles and a 600 °C thermal treatment in air, in order to compare the hybrid structure to an inorganic one. Polymer film thickness and sample substrate were chosen based on each characterization technique’s requirements and are detailed in Table 2 in the experimental section.

As mentioned above, we deciphered the crystal structure of hybrid polymer-ZnO_*x*_ films by XANES and HR-STEM. Synchrotron-based Zn K-edge XANES provided statistically significant information regarding ZnO_*x*_ local atomic structure, including Zn–O and Zn–Zn coordination numbers and distances in the first and second coordination shells of the absorbing zinc atoms, respectively. High angle annular dark field (HAADF) STEM enabled direct, atomic scale imaging of ZnO_*x*_ crystals while still in the polymeric matrix, which provided vital information on particle size, dispersion in the polymer, and corroboration of the crystal structure model obtained from the XANES data.

### 1.1 The Effect of Polymer Chemistry on ZnO_*x*_ Growth

**Figure 2:**
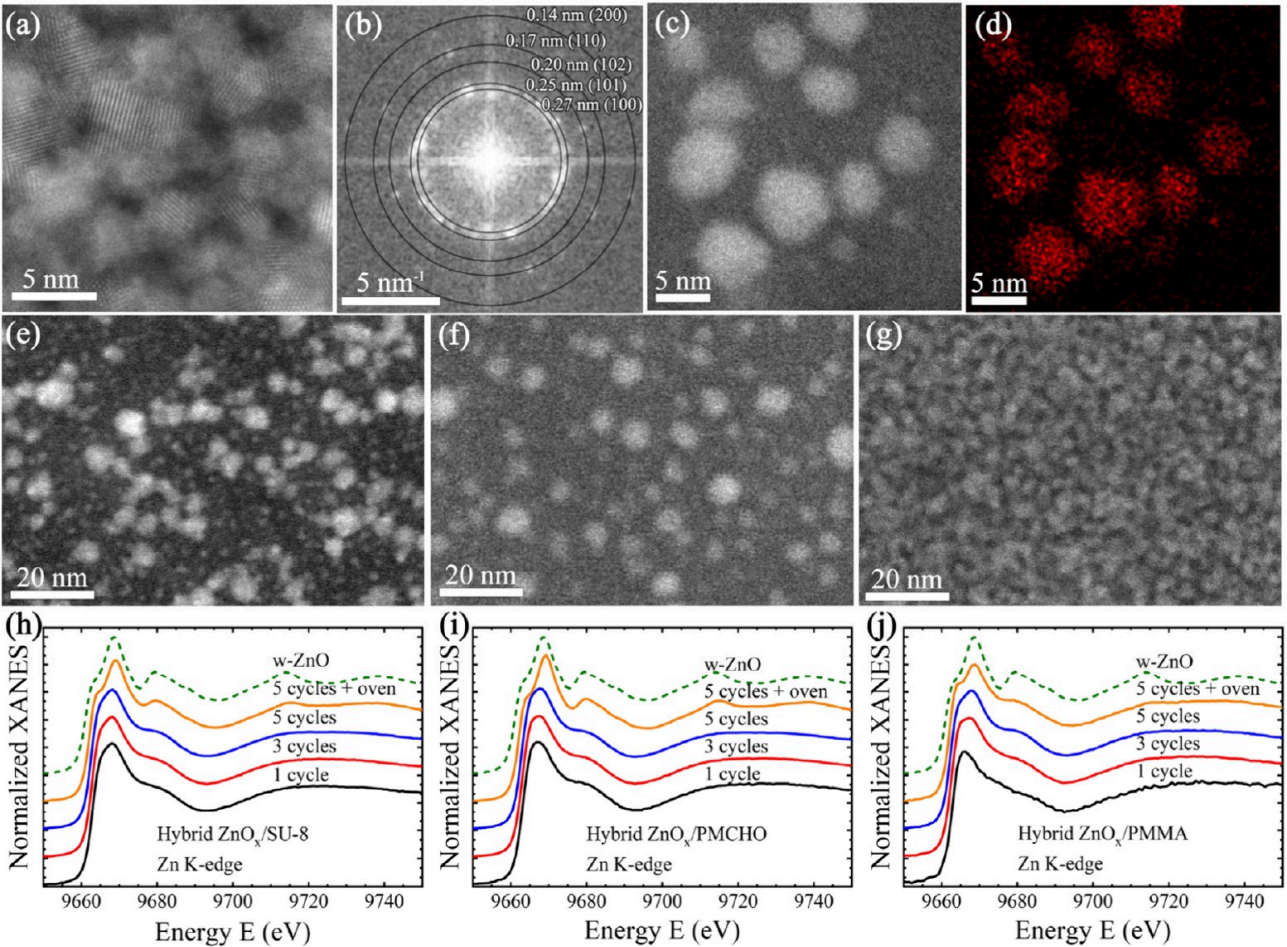
ZnO_*x*_ growth in polymers: (a) atomic resolution STEM of hybrid ZnO_*x*_/PMMA film and (b) its corresponding FFT analysis; (c) STEM of hybrid ZnO_*x*_/PMCHO film and (d) its corresponding STEM-EDX Zn mapping; hybrid polymer–metal oxide films after 5 cycles of DEZ in (e) SU-8, (f) PMCHO and (g) PMMA films; (h-j) Zn K-edge XANES spectra for hybrid ZnO_*x*_/SU-8, ZnO_*x*_/PMCHO, and ZnO_*x*_/PMMA after 1, 3, and 5 cycles, as well as after 600 °C thermal treatment in air, with a comparison to pure ZnO Wurtzite (dashed lines).

We performed HR-STEM on the hybrid film to reveal the atomic structure of ZnO_*x*_, as grown by the SIS process (Figure 2). The growth resulted in polycrystalline ZnO_*x*_ particles, intimately embedded within the polymer films, with the wurtzite-type structure – *P*6_3_mc. The atomic structure was determined through analysis of the fast Fourier transform (FFT) of STEM images taken at each polymer-ZnO_*x*_ system. For example, Figure 2b shows the FFT analysis of Figure 2a, hybrid ZnO_*x*_/PMMA after 5 SIS cycles. Periodicities detected in the image were measured as representing diffraction from the interplanar spacings of 2.7, 2.5, 2.0, 1.7, and 1.4 Å, corresponding to the wurtzite-type *d*-spacings of 2.81 (100), 2.48 (101), 1.91 (102), 1.62 (110), and 1.40 Å (200), respectively (FFT analysis of hybrid ZnO_*x*_/SU-8 and hybrid ZnO_*x*_/PMCHO can be found in Figure S1). The correlation between HAADF STEM bright regions and Zn atoms was determined by STEM-energy dispersive X-ray spectroscopy (STEM-EDX), as shown in Figure 2c and d, of hybrid ZnO_*x*_/PMCHO after 3 cycles. A similar analysis was performed in SU-8 and PMMA (Figure S2), corroborating that the bright contrast in HAADF STEM images originated from ZnO_*x*_.

ZnO_*x*_ particle sizes and densities within the polymer significantly differed between the different polymer chemistries. In SU-8 and PMCHO, 5 cycles resulted in discrete particles, with some diversity in particle size and overall coverage, as measured by image analysis. Particles in SU-8 (Figure 2e) were composed of two particle size populations: larger particles of 5.8 nm with 1.3 nm standard deviation (SD), and smaller 2.2 nm with 0.6 SD (combining the two populations results in an average size of 4.2 nm with 2.0 SD). High-intensity areas, which correspond to ZnO_*x*_ (as shown by EDX), cover ∼30% of the SU-8 imaged area. PMCHO (Figure 2f) has similar, 30% coverage, with more uniformly sized particles of 5.8 nm average diameter and 1.4 nm SD (the same as the larger particles in SU-8). PMMA differs from the other two polymers since its preseeding step highly encourages growth. After 5 cycles, particles in PMMA are so abundant that they appear interconnected (Figure 2g). They cover 70% of the imaged area, and their average size (gleaned by measuring separate grains) is 4.3 nm with 1.0 SD, smaller than PMCHO and SU-8 particles, suggesting a higher number of nucleation sites during initial growth. The Zn K-edge XANES spectra (Figure 2 h-j) are quite similar and suggest a very small size of ZnO_*x*_ clusters,^57^ as their shape is characteristic of a Zn environment composed of mainly 4 oxygen atoms in the first coordination shell, with some weak contribution from zinc atoms in the second coordination shell which increases slightly with the number of cycles.^58^ After 5 cycles and 600 °C thermal treatment in air, the shape of the XANES spectra becomes closer to that of the ZnO bulk (dashed line) as the contribution from the neighboring Zn atoms located in the second coordination shell appears stronger at 9680 eV.

There are several factors which may contribute to the observed differences in ZnO_*x*_ growth between the polymers. The most notable factor is the polymers’ different functional groups, or their chemistry. This factor can affect precursor sorption, diffusion, and polymer-precursor interactions, which are crucial for reaction. Another factor noted in past works is the choice of solvent, which may induce a reaction in an otherwise non—reactive polymer.^25^ In this work, we used cyclopentanone to spin-coat SU-8, and toluene for PMMA and PMCHO. Additionally, in SU-8 and PMCHO, under certain conditions, there may be variations in the functional groups. In many studies of ZnO growth within SU-8, the polymer is UV cross-linked prior to SIS, making the epoxide groups no longer available for reaction, with ethers in their stead. Here, we took no deliberate cross-linking step, so the epoxides are still available, but since they are extremely reactive groups, it is highly probable that a certain percentage has been cross-linked nonetheless, through exposure to process heat (120 °C) and UV light from the environment. There are, therefore, three potential moieties for interaction in SU-8: unreacted epoxides, reacted epoxides, and cyclopentanone (solvent) molecules. Each such moiety might facilitate a different reaction with its own growth mechanism. In PMCHO, upon exposure to humidity, the aldehyde might undergo hydration, resulting in the corresponding acetal, *i.e.*, two hydroxyl groups in the carbonyl’s stead. These variations from the original molecules may introduce differences in growth mechanisms between the polymers, as well as within each polymer. Finally, since DEZ is known to be extremely reactive with water, any presence of water residues inside the films could result in a chemical vapor deposition (CVD)-like reaction, where DEZ interacts with water molecules, without direct interaction between the precursor and the polymer moieties.

In order to determine which factor is dominant in these studied systems, we conducted *in situ* FTIR spectroscopy measurements. The measurements, which are taken every 30 s in reflectance mode, provide insights into the reactions happening within the polymer films and in the ALD chamber. The interaction between PMMA and the first precursor, TMA, has already been studied by *in situ* FTIR.^7^ Trends seen in this study during the first cycle are similar (Figure S3, Table S1), where a portion of the peak corresponding to the carbonyl (1738 cm^–1^) is shifted to a lower wavenumber (1668 cm^–1^), indicating the weakening of the C=O bond due to C–O–Al bond formation. Also evident is precursor C–H stretch around 3000 cm^–1^, Al–C interaction (700 cm^–1^) and an increase in AlO_*x*_ signal (<1000 cm^–1^) after the first water pulse, compared to the baseline. In all DEZ exposures, on the other hand, only the ∼3000 cm^–1^ C–H stretch is evident while other changes in polymer moieties, if existing, are below measurement sensitivity. Similarly, in SU-8 (Figure S4, Table S2), all peaks that correspond to the aforementioned functional groups remain unchanged during the process, including epoxy ring C–O–C bend (915 cm^–1^) and stretch (832, 1248.5 cm^–1^), ether groups (1037–1184 cm^–1^) and the cyclopentanone (solvent) carbonyl C=O stretch (1735.5 cm^–1^). In fact, the only evident change is an increase in precursor C–H stretch at 2930 cm^–1^, which is completely reduced during purge times. It is clear, however, that with advancing cycles, there’s an increase in the 1037 cm^–1^ peak (corresponding to ether groups) during water exposure, hinting at an increase in water uptake following ZnO_*x*_ nucleation. Another change is a near-linear increase around 660 cm^–1^, corresponding to the ZnO_*x*_ signal, as well as a similar increase in O–H 3436 cm^–1^ beginning from the fourth cycle water pulse, which could indicate the presence of hydroxyl groups on larger ZnO_*x*_ particles, as expected from ALD chemistry. Finally, in PMCHO (Figure S5, Table S3), as in SU-8, the only evident change over time is the appearance and disappearance of DEZ C–H stretch at 3020 cm^–1^ during precursor exposure times, with no change to aldehyde C=O stretch peak (1678, 1752.8 cm^–1^), acetal C–O–H stretch peak (1152.9, 1194 cm^–1^) and a slight increase in <1000 cm^–1^ region corresponding to ZnO_*x*_ growth.

The apparent lack of DEZ interaction with both polymer and solvent functional groups according to FTIR measurements cannot entirely rule out the existence of such interactions, which could be too small to be noticeable within the method’s sensitivity limits. It is, however, unlikely that these interactions are the dominant mechanism for growth, since we would then expect to see greater change, more similar to the changes seen in PMMA/TMA reaction. One hypothesized mechanism of which we cannot directly learn from FTIR measurements is the CVD-like growth mechanism. During SIS experiments, it is generally assumed that in the conditions used in the ALD chamber of high temperature and high vacuum and during sufficiently long purge steps, all water molecules leave the polymer or hybrid film. However, as is the case with leftover solvent molecules, so it can be with water, where the drying steps performed before the experiment (outside and within the ALD) are insufficient to truly rid the film of all water residues. In that case, a polymer or a hybrid material that is more hygroscopic would have a higher water content during the SIS process (*i.e.*, before the beginning of each cycle, following either drying in the first cycle or water purge in the following ones), which could account for the difference in crystal evolution between the polymers. The possibility of a reaction with water molecules will be further discussed in the following sections.

### 1.2 From Hybrid Material to Inorganic Structure

**Figure 3:**
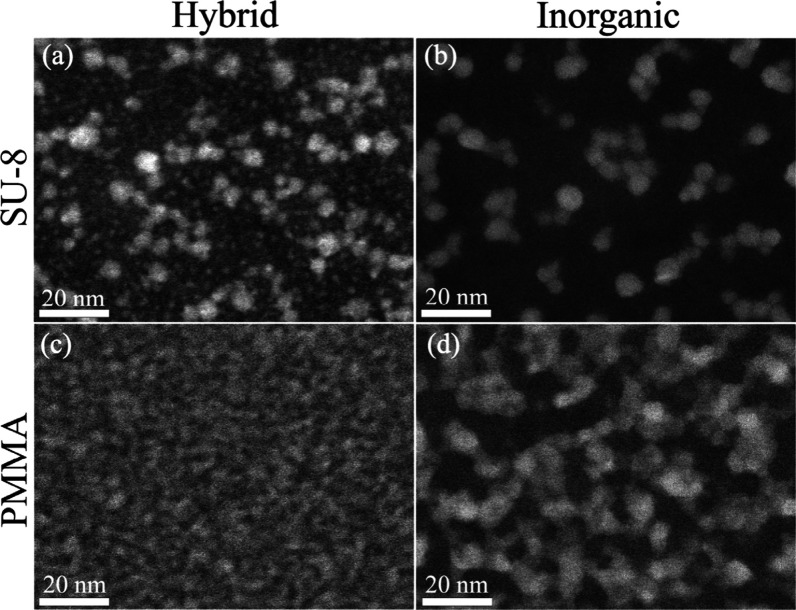
Comparison between hybrid polymer–metal oxide (a, c) and inorganic (b, d) films. (a, b) are STEM images of SU-8 samples; (c, d) are PMMA samples. Inorganic films were attained by burning their hybrid counterparts at 600 °C in air. PMMA sample underwent 1 cycle of AlO_*x*_ SIS prior to DEZ infiltration, to encourage growth. Scale bars are 20 nm.

To study the transformation from hybrid film into inorganic structure upon polymer removal, we performed HR-STEM and EXAFS analysis on film prior to and after thermal treatment. High temperature (600 °C) exposure to oxygen resulted in both polymer removal and sintering of ZnO_*x*_ nanocrystals. It allows migration of small crystals which then combine into larger ones. In the sparsely grown ZnO_*x*_ in SU-8 (Figure 3a, b) the two size populations observed in the hybrid film (Figure 3a) form 7.2 nm particles with 1.6 nm SD after thermal treatment (Figure 3b). Burning of the more densely grown PMMA-ZnO_*x*_ film (Figure 3c, d) results in what appears to be a network-like structure composed of individual particles with 6.4 nm average diameter and 1.9 nm SD (Figure 3d). FFT analysis of images exhibiting lattice fringes both before (SU-8 in Figure S1 and PMMA in Figure 2) and after (Figure S6) thermal treatment shows that crystals grown in both polymers maintain their wurtzite-type structure, as also demonstrated from EXAFS spectra fits and data in Table S4. Fourier transforms (FTs) of the Zn K-edge EXAFS spectra are shown in Figure S7.

### 1.3 Growth Evolution with SIS Cycles

**Figure 4:**
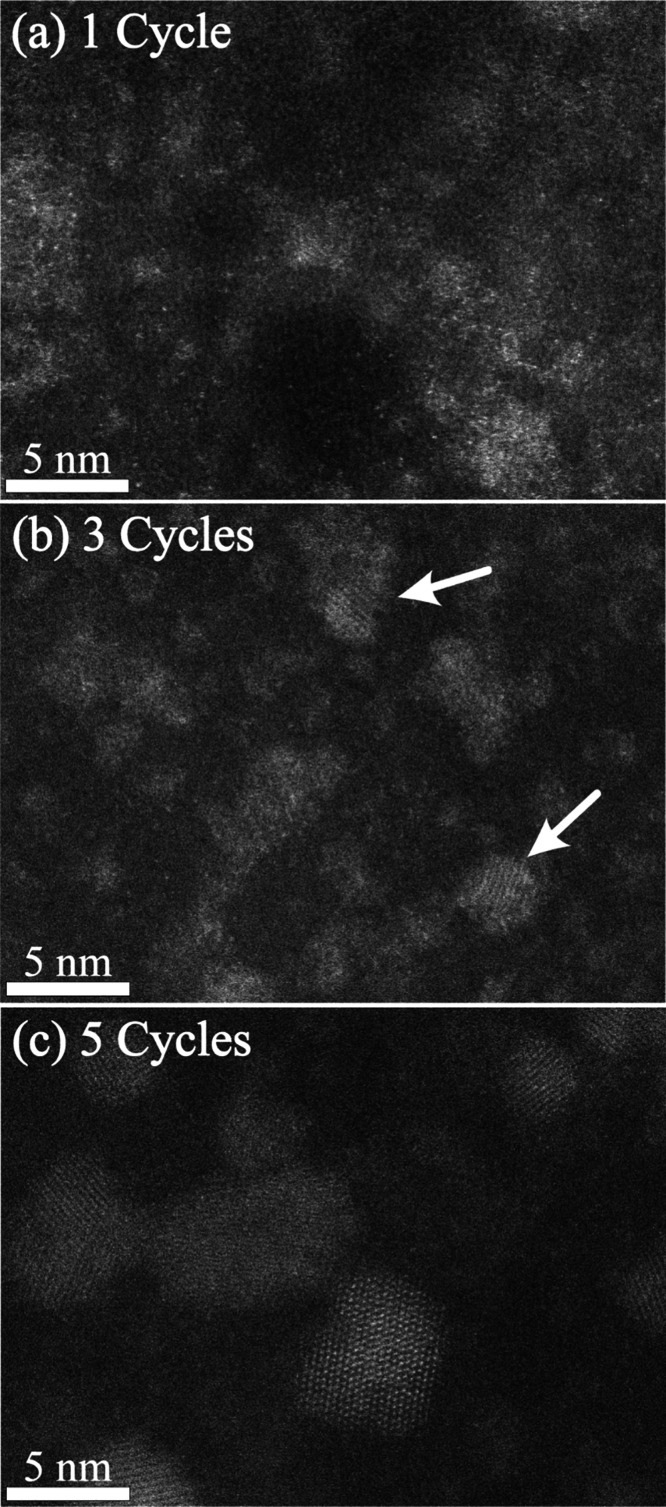
Growth evolution with cycles in SU-8: atomic resolution STEM images of hybrid ZnO_*x*_/SU-8 after 1 (a), 3 (b), or 5 (c) cycles.

**Figure 5:**
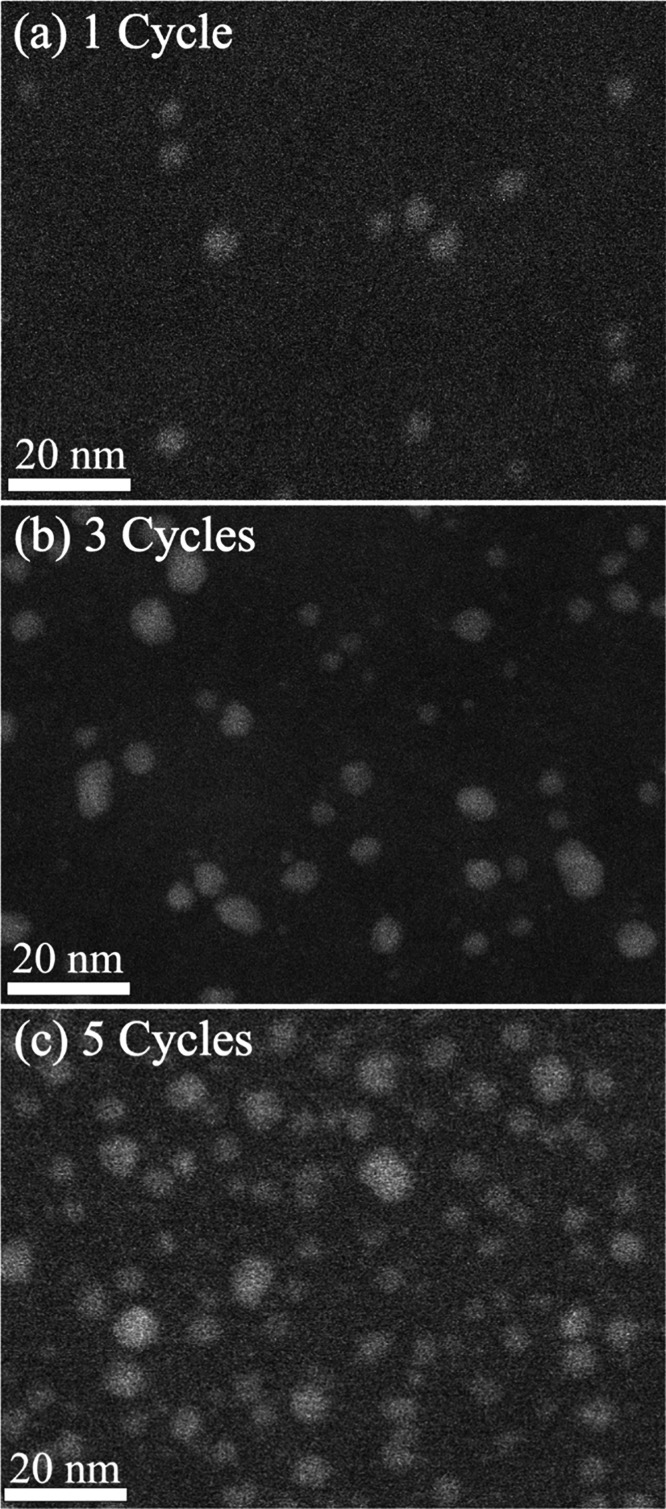
Growth evolution with cycles in PMCHO: STEM images of hybrid ZnO_*x*_/PMCHO after 1 (a), 3 (b), or 5 (c) cycles.

**Figure 6:**
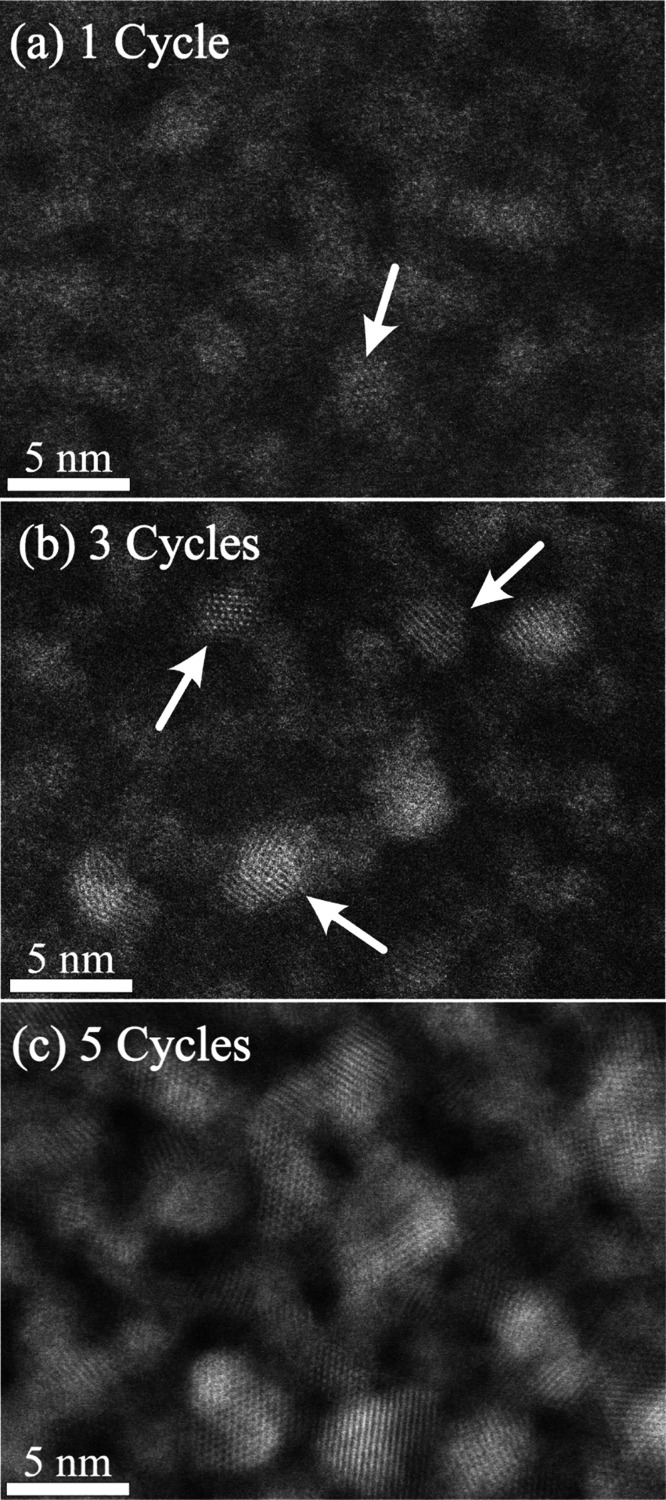
Growth evolution with cycles in PMMA: Atomic resolution STEM images of ZnO_*x*_ after 1 (a), 3 (b), or 5 (c) cycles. one cycle of AlO_*x*_ preseeding was done in all three samples.

In polycrystalline growth via SIS, there are several unanswered questions. The major ones are the following: When does the crystalline structure start to form? How does it evolve? These questions and insights into the different crystal growth in the three polymers can be gleaned from studying the crystal evolution with SIS cycles. A full comparison of the growth evolution in SU-8, PMCHO, and PMMA using EXAFS (Table 1 and Figure S8) and HR-STEM (Figures 4–6, Figure S9) reveals tremendous differences between the polymers. In all three polymers, particle size and their density within the polymer grow with cycles. However, the nucleation density, growth rates, and the level of ZnO_*x*_ crystallinity at early growth cycles significantly differs between the polymers. In SU-8 and PMMA, the crystals form in a gradual process, as evident by the low number in the second shell (*N*_Zn–Zn_, Table 1) after a single cycle, and its increase with cycles to 12, which corresponds to a fully ordered wurtzite structure. PMCHO, on the other hand, displays fully ordered wurtzite structure already after a single SIS cycle. To gain better understanding of the ZnO_*x*_ crystal formation and evolution, we turn to atomic resolution STEM imaging.

**Table 1:** Structural Parameters for the First and Second Coordination Shells of Zn Obtained from the Fitting of the Zn K-Edge EXAFS Spectra^a^

		**SU8**			**PMCHO**			**PMMA**		
	Number of cycles	1	3	5	1	3	5	1	3	5
Zn–O	*N*	4.0	3.8	3.5	3.7	2.9	3.6	4.0	3.7	3.7
	*R*	2.02	2.02	2.01	2.01	2.01	2.01	2.02	2.01	2.02
	MSRD	0.010	0.008	0.005	0.008	0.007	0.007	0.009	0.007	0.007
	*C*_3_	0.001	0.001	0.001	0.001	0.001	0.001	0.002	0.001	0.002
Zn–Zn	*N*	**2.3**	**5.2**	**12.0**	**12.0**	**12.0**	**12.0**	**8.4**	**12.0**	**12.0**
	*R*	3.33	3.45	3.44	3.38	**3.53**	3.46	3.33	3.48	3.44
	MSRD	**0.022**	**0.026**	**0.039**	**0.043**	**0.036**	**0.039**	**0.039**	**0.037**	**0.034**
	*C*_3_	0.000	0.005	0.006	0.000	0.012	0.007	0.002	0.008	0.006

a Here *N* is a coordination number (±0.4), *R* is an average interatomic distance, MSRD is mean-square relative displacement (σ^2^, ± 0.002 Å^2^), also known as the Debye–Waller factor, and *C*_3_ is a third cumulant (±0.0005 Å^3^) which accounts for a deviation of the radial distribution function from the Gaussian shape.

Atomic resolution STEM imaging of SU-8 after 1, 3, or 5 cycles is presented in Figure 4. To the best of our knowledge, this is the first imaging of nucleation points and clusters after a single SIS cycle. We observed relatively dispersed growth after this first cycle (Figure 4a), with amorphous cloud-like bright regions and very small ZnO_*x*_ clusters with no clear crystal structure. This nebulous growth was corroborated by the analysis of the EXAFS spectrum (data in Table 1), which shows that after a single SIS cycle, the coordination number of zinc in the second shell was 2.3. The low *N* observed after a single SIS cycle indicates a low level of crystalline formation, in agreement with the STEM data. After two additional cycles (Figure 4b), small crystals were evident (white arrows), and *N* increased to 5.2. As seen in lower magnification STEM (Figure S9c), as well, 5 cycles led to clear, well-formed crystals with the number of zinc atoms in the second coordination shell equal to 12, indicating fully ordered wurtzite structure. This analysis shows that in SU-8, the first cycle forms amorphous nucleation points and clusters within the polymer film, which then rather quickly develop into discrete and ordered polycrystalline particles with progressing cycles.

In contrast to SU-8, in PMCHO after a single SIS cycle, the number of zinc atoms in the second coordination shell is already equal to 12, the coordination number of zinc in fully ordered wurtzite crystal (Table 1), and remains constant following additional cycles, indicating that crystalline particles were already formed at the first cycle. We note that due to PMCHO’s sensitivity to the electron beam, lower magnification images were obtained. With advancing cycles (Figure 5), average particle size grows from 4.8 nm with 0.8 nm SD, through 4.9 nm with 1.3 SD and finally 5.8 nm with 1.4 SD, for 1, 3, and 5 SIS cycles, respectively. Though similar in average size, the different SD between 1 and 3 cycles pronounces the change in particle size diversity between the two samples, where after 1 cycle particles are relatively uniform in size, but after 3 cycles two size populations are evident, similarly to SU-8. These two populations are also evident after 5 cycles, where the SD is similar to 3 cycles, but larger particles become more abundant, as demonstrated by the larger average diameter. The overall trend between the three PMCHO samples is an increase in the number of particles per area unit, suggesting the nucleation of new particles with each cycle, as well as existing particle growth. The formation of new particles with advancing cycles, their high crystallinity level, as well as the rapid rate of growth, differ from growth trends expected in ALD-like growth. A possible explanation for this difference will be discussed in the following section.

Finally, we examine the interesting system of ZnO_x_ growth within PMMA with AlO_*x*_ preseeding step (Figure 6). Here, the ZnO_*x*_ cycle evolution differs from the other two polymers due to the AlO_*x*_ preseeding step, which creates high-density AlO_*x*_ clusters bound to the PMMA.^7,40^ Indeed, after just a single ZnO_*x*_ SIS cycle (Figure 6a), we already observed the onset of crystallinity with small crystals formation (white arrow) and a second shell coordination number *N* of 8.4 (Table 1). After 3 cycles, the level of order significantly increased, with a higher concentration of small crystals (Figure 6b), and a second shell coordination number of 12, *i.e.*, fully ordered wurtzite crystals were formed. After 5 cycles (Figure 6c), as previously mentioned, particles become interconnected, and the second shell coordination number *N* remains 12. The HR-STEM and the EXAFS data have excellent agreement and show the fast evolvement of highly dense ZnO_*x*_ crystals in PMMA with AlO_*x*_ preseeding step.

## Discussion

**Figure 7:**
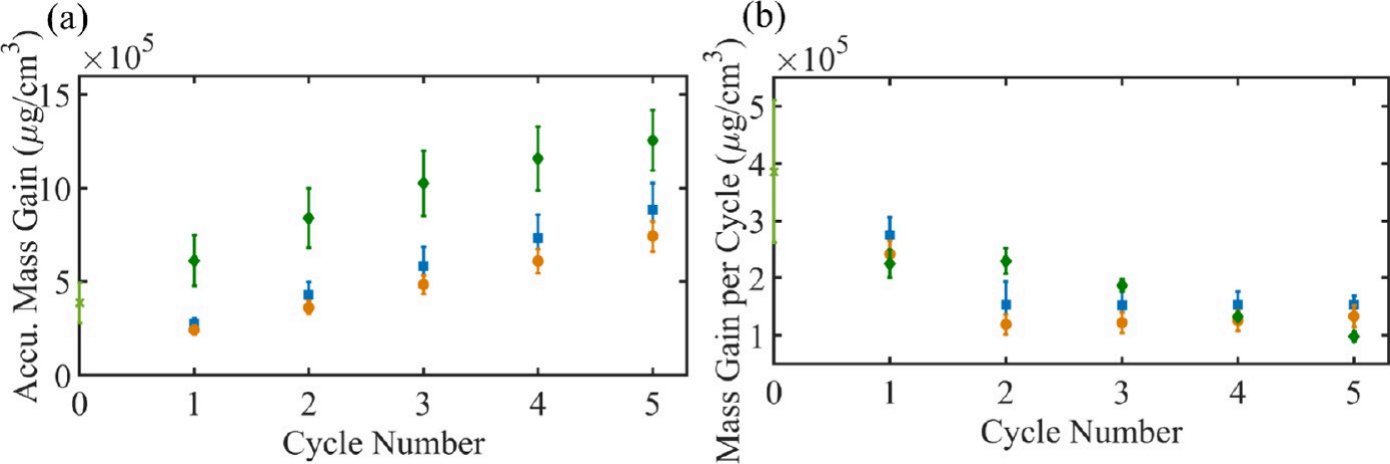
QCM measurements of accumulated mass (a) and mass gained after each cycle (b) for SU-8 (blue square), PMCHO (orange circle) and PMMA (green diamond). Green stars represent mass gained during the TMA cycle in PMMA films.

To gain better understanding of the growth mechanism, we also probed the cycle evolution using two *in situ* methods, in addition to the *ex situ* methods- XAS and HR-STEM. The first, *in situ* FTIR, which provides insight into chemical interactions, was already discussed in the previous section. The second method, *in situ* microgravimetric analysis, enabled us to probe mass gain during each step and cycle of the process (Figure 7 and Figure S10). The mass gained during the first cycle is the highest in all polymers, but the trend seen in subsequent mass gain differs. While in PMCHO and SU-8 mass gain is approximately constant with cycles, in PMMA there is a decline with each additional cycle. This difference is due to the overall amount of metal-oxide grown in each system. Since the direct reaction between PMMA side chains and TMA results in high-density AlO_*x*_ seeding, this well-dispersed AlO_*x*_ greatly encourages subsequent ZnO_*x*_ growth in PMMA. The polymer film therefore becomes saturated with increasing cycles (as seen in Figure 6c), and this reduces the free volume for diffusion into the film, similarly to research done in AlO_*x*_ SIS in PMMA.^59,15^ In PMCHO and SU-8, on the other hand, due to the sparse, discrete nucleation, even after several cycles, there is still ample volume for diffusion, allowing for a constant rate of mass gain.

**Figure 8:**
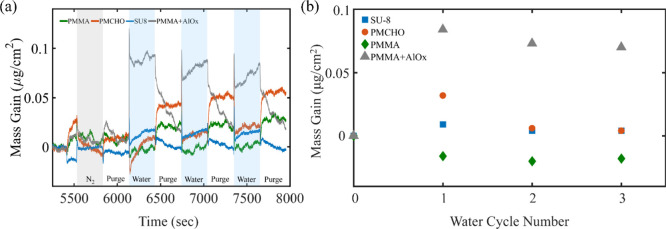
QCM water uptake evaluation: raw data (a) and mass gain per cycle (b) for each polymer, as well as for the PMMA/AlO_*x*_ hybrid. Experiments were done at 120 °C. Each cycle was comprised of 300 s exposure to water, followed by 300 s N_2_ purge. Before the first exposure to water, a single cycle was performed without opening the water valve, to help eliminate N_2_ and chamber valve procedure artifacts (cycle 0, gray area in (a)). Cycle 0 values were reduced from all mass gain values. Exposures were done in static mode, during which the chamber is completely closed and there is no N_2_ flow.

In order to explain the growth evolution in these polymers, it is necessary to distinguish between growth patterns in the first cycle, where the polymer is in its pristine state (for PMCHO and SU-8), and the following cycles, where there is already the presence of a metal-oxide. As mentioned in the previous section discussing FTIR, at the first DEZ exposure, the most likely growth mechanism is a reaction between the DEZ and water molecules that are present in the polymer film. To probe the likelihood of this hypothesis, we conducted microgravimetric measurements, where extremely dry polymer films were repeatedly exposed to water vapors and then purged in order to evaluate their hygroscopic nature (Figure 8). This delicate experiment requires highly stable setup and is described at length in the SI, with pressure profile, flow and valve positions detailed in Figure S11e. Figure 8a depicts mass gain during a water uptake experiment for each polymer (PMMA, PMCHO and SU-8 in green, orange and blue, respectively), as well as for a PMMA/AlO_*x*_ hybrid, after a single TMA/H_2_O cycle (gray). For clarity, Figure 8a plots are also presented in Figure S11a-d on separate axes. Figure 8b shows the mass gain per half cycle calculated from the plots in Figure 8a (the difference between the mass at the end of the exposure to water and the mass just prior to its beginning). The average value measured in the control step, during which the chamber was only exposed to N_2_ at 5 sccm (gray half cycle), was reduced from all values measured in both the N_2_ and water cycles in Figure 8b. It is noticeable that mass gained in all PMMA and SU-8 half cycles is approximately constant, while in PMCHO and in the hybrid films, a decrease is seen between the first and following half cycles. Another visible trend is the difference in water mass gain between the polymers, where during the first exposure, PMMA exhibits the lowest mass gain (negative value, interpreted as zero mass gain), followed by SU-8 (0.009 μg/cm^2^), PMCHO (0.032 μg/cm^2^), and PMMA/AlO_*x*_ hybrid with the highest mass gain (0.084 μg/cm^2^). During these first exposures, water mass gain in the PMMA/AlO_*x*_ hybrid was 2.5 times higher than that of PMCHO; PMCHO water uptake was 3.5 times higher than that of SU-8, and pristine PMMA’s water uptake was negligible. We attribute the difference in hygroscopy to the polymers’ functional groups, which are responsible for forming hydrogen bonds with water molecules, as well as to the difference in polymer chain length, which greatly affects a polymer’s solubility in a given solvent. The increase in mass upon exposure to nitrogen, as seen in PMCHO and PMMA, may be due to nitrogen absorption or sensitivity of the system to the opening and closing of the valves.

The difference between the first and following half cycles can be explained by a closer look at the experiment dynamics. While in SU-8 and PMMA mass is approximately constant during purge times, in PMCHO and PMMA/AlO_*x*_ hybrid, mass decreases significantly at each purge, indicating continued water removal from the film that does not plateau before the following half cycle. Water removal in them is slower than the other polymers, and they remain highly hydrated at the beginning of the following half cycle, reducing further water uptake in the next one. Since water diffusion is extremely fast at high temperatures, and with constant evacuation using the carrier gas at low pressure, it is generally assumed that during purge all the water leaves the film. However, since DEZ is extremely sensitive to water presence, even traces are sufficient to facilitate the reaction. A higher presence of water in the hybrid material, left over from the TMA/H_2_O cycle, explains the significantly higher ZnO_*x*_ growth witnessed in PMMA/AlO_*x*_. PMCHO, next on the water uptake values, shows the highest growth during the first SIS cycle, followed by SU-8, and finally, little to no growth in pristine PMMA. Similarly to AlO_*x*_, growth of ZnO_*x*_ increases the films’ hygroscopy, resulting in increased growth in the following cycles.

Overall, the hygroscopy levels of the pristine polymers (and hybrid PMMA/AlO_*x*_) can be summarized as PMMA/AlO_*x*_ > PMCHO > SU-8 > PMMA. This trend correlates well with growth patterns shown by HR-STEM and EXAFS. ZnO_*x*_ mass gain in PMMA is high after the first DEZ/H_2_O cycle, but with many nucleation sites introduced by the presence of AlO_*x*_ seeding, grown particles are relatively small. In PMCHO, high hygroscopy likely results in high water aggregates within the film, which lead to few, but large and well-defined, ZnO_*x*_ crystals. SU-8’s relatively low hygroscopy allows for nucleation, but scarce and nebulous growth, and finally, pristine PMMA’s negligible hygroscopy does not allow for any ZnO_*x*_ growth.

Another important observation from these measurements is that in the hybrid film, the time allowed for water purge during the SIS process is insufficient to completely rid the system of water and return to the baseline. Though purge times used in these water experiments were shorter than those used in ZnO_*x*_ growth experiments, it was noticeable that stabilization times required to reach completely dry films were long, namely hours rather than minutes. It is therefore highly probable that some water molecules remain after each water half cycle used to grow ZnO_*x*_, yielding increased growth rate and deviating from the self-limiting, ALD-like behavior of SIS and behaving more as a CVD reaction. Thus, after the initial metal oxide nucleation within the polymer, the growth varied between two possible growth mechanisms: ALD- and CVD-like growth. In ZnO ALD performed on clean Si wafer and Au-coated quartz crystal at 120 °C, the growth per cycle (GPC) during the constant growth rate region was 1.71 Å. Applying this number to a spherical system should result in 0.342 nm increase in sphere diameter per cycle, 0.684 nm after two cycles, and 1.71 nm after 5 cycles. In PMCHO, average particle diameter grew in 0.1 nm from 1 cycle to 3 cycles (with a large increase in SD, indicating the growing variety in diameters), and then in 0.9 nm from 3 to 5 cycles. A similar analysis in SU-8 and PMCHO was unavailable due to the small diameter and sparse growth seen after 1 and 3 cycles. These numbers alone allow for the possibility of ALD-like growth, but they cannot account for the overall average diameters of 4.2, 4.3, and 5.8 nm for SU-8, PMMA, and PMCHO respectively, which is almost three times higher than the total 1.71 nm at the highest rate of ALD-like growth. We, therefore, hypothesize that during the first cycle, growth is entirely CVD-like in nature, wherein DEZ reacts with water or leftover solvent molecules, while in the following cycles, a combination of both methods occurs, with new nucleation sites forming mostly by DEZ reaction with water molecules, but also with hydroxyl groups present on the surface of previously formed particles. Similarly to nonsufficient purge times that hinder the self-limiting nature of ALD reactions, polymer hygroscopy and the required prolonged purge times, may reduce the self-limiting nature of SIS.

Observations gleaned in this research could have far-reaching implications for the expansion of SIS studies to new polymers and new precursors. While past research implies that water presence does not play a role in the classic TMA-PMMA reaction, the same cannot be assumed for any future polymer-precursor pairings. Each pairing must be extensively studied in order to ascertain that the reaction occurring in the polymer is indeed self-limiting and reveal the nature of the chemical bond formed during the first exposure step. During the following cycles, we should bear in mind the possibility that even if the first cycle was self-limiting, the following ones are not necessarily so. The presence of metal oxide in the polymer fundamentally changes its chemical properties, increasing its hygroscopic nature, and thus making the proper evacuation of water during the purge step infinitely more challenging. Yet, though troublesome when a self-limited reaction is desired, this increased water absorption could also be harnessed to increase overall mass gain, by encouraging the CVD-like reaction to occur through the addition of a deliberate water-saturating step. In either case, an understanding of the true growth mechanism is imperative for the judicial choice of reaction conditions in all studied systems.

## Conclusions

Using a combination of EXAFS and HR-STEM, we examined the interaction of three representative polymers- PMMA, SU-8, and PMCHO, with the organometallic precursor DEZ, resulting in the growth of polycrystalline zinc oxide particles within the polymer films. By studying zinc oxide nanocrystals in their native polymeric environment, we found that their crystal structure in all three systems is wurtzite, both before and after polymer removal *via* thermal treatment. Through *in situ* FTIR analysis, we observed that DEZ molecules have little to no direct interaction with the polymer moieties, reacting instead with water residues inside the films. Differences in water absorption affect ZnO_*x*_ growth during the first SIS cycle, resulting in no growth in pristine PMMA, nebulous growth in SU-8, distinct particles in PMCHO, and small, yet abundant, crystals in the hybrid AlO_*x*_/PMMA, in correlation to the polymers’ water absorption trends. Additional cycles are hypothesized to occur in a similar manner, with water residues from the previous cycle reacting with the current cycle’s DEZ, bringing about both the formation of new ZnO_*x*_ nuclei and the growth of existing particles. In addition, it is assumed that a classic ALD-like reaction occurs concurrently, in which DEZ interacts with hydroxyl groups located on the surface of previously formed ZnO_*x*_ crystals. This insight into the growth of ZnO_*x*_ in polymers is important for future study of these, and other systems. Scrupulous drying of films prior to experiments could help avoid a CVD-like reaction, while deliberate and judicious exposure to water vapors could encourage significant ZnO_*x*_ growth. Similarly, hydrophilic polymers have the potential to facilitate larger mass gain, while more hydrophobic polymers could lead to self-limiting reactions, similar to those generally assumed in SIS.

## Methods

### 1.4 Materials

PMMA (*M*_n_: 105.7 kg mol^–1^, PDI 1.8) and PMCHO (*M*_n_: 1.5 kg mol^–1^, PDI 1.2) were purchased from Polymer Source. SU-8 2002 in cyclohexanone was purchased from Microchem. Organometallic precursors trimethyl aluminum (TMA) and diethyl zinc (DEZ) were purchased from STREM. Unpolished gold-plated quartz crystals (SC-101, nominal resonant frequency 6 MHz) were purchased from Inficon. Si wafers, 4″, 200 μm thick, double-sided polished and coated with 30 nm super low-stress SiN_*x*_ (SiN_*x*_:Si:SiN_*x*_ – 30 nm:200 μm:30 nm) were purchased from Pure Wafers. All materials were used as received.

### 1.5 Sample Preparation

Polymer films were spin-coated onto different substrates, depending on the application, as listed in Table 2. PMMA and PMCHO were spin-coated from toluene; SU-8 was spin-coated from cyclohexanone. Following spin-coating, films were relaxed on a hot plate at 60 °C for 30 min in air and then kept in a low humidity environment for at least 2 h prior to SIS.

**Table 2:** Sample Description

**Method**	**Substrate**	**Film Thickness**
EXAFS	Glass slides	∼300 nm
QCM	Gold-plated quartz crystals	50 nm
HR-STEM	SiN_*x*_ TEM windows	15–20 nm
FTIR	Au-coated Si wafers	∼300 nm

SiN_*x*_ TEM windows preparation was as follows: back side SiN_*x*_-Si-SiN_*x*_ wafers were patterned with an array of 1 mm × 0.2 mm rectangles with 3 mm spacing, using optical lithography and reactive ion etching (RIE) plasma (Thermo 790, CHF_3_ - 36 sccm and O_2_ - 4 sccm, 40 mTorr, RF power 175 W, 20 °C), to serve as an etch mask. After polymer spin-coating onto the front side and SIS, the back side was wet-etched in 30 wt % KOH aqueous solution at 95 °C, with the front side protected, resulting in rectangular 30 nm thick SiN_*x*_ windows. Inorganic films were attained by burning their hybrid counterparts at 600 °C in air.

### 1.6 Sequential Infiltration Synthesis

SIS was done in a commercial ALD system (Savannah S100, Veeco). Polymer films coated on the different substrates were placed in the ALD chamber at 120 °C, with 20 sccm N_2_ flow and 0.3 Torr base pressure. Precursors were kept at room temperature. SIS consisted of 5 consecutive cycles. During each cycle, the DEZ valve was opened for 0.015 s, and precursor vapors were carried into the chamber by 5 sccm N_2_ flow. The chamber was fully sealed for 900 s exposure and then opened for 1200 s N_2_ purge (20 sccm) to evacuate excess precursor. Exposure and purge steps were then repeated for the H_2_O coreactant. In PMMA films, 5 DEZ cycles were preceded by a single TMA cycle, done under the same conditions. *In situ* crystal microgravimetry experiments were done using a dedicated chamber lid as previously reported.^15^

### 1.7 Extended X-ray Absorption Fine Structure

X-ray absorption spectroscopy experiments were performed at room temperature in fluorescence mode at the Zn K-edge using a passivated implanted planar silicon (PIPS) detector at the P65 Applied XAFS beamline^60^ of the PETRA III storage ring. The storage ring operated at energy *E* = 6.08 GeV and current *I* = 100 mA in top-up 40 bunch mode. The harmonic reduction was achieved by an uncoated silicon plane mirror. The X-ray beam from an undulator was monochromatized using fixed-exit double-crystal Si(111) or Si(311) monochromators. The intensity of the incoming X-ray beam was measured by an ionization chamber.

Experimental X-ray absorption spectra were treated using the XAESA code.^61^ Both X-ray absorption near edge structure (XANES) and extended X-ray absorption fine structure (EXAFS) parts were extracted. The analysis of EXAFS spectra was performed using the conventional EXAFS equation within the single-scattering approximation.^62^ The contributions from the first and second coordination shells of zinc, composed of oxygen and zinc atoms, respectively, were isolated by the Fourier filtering procedure in the *R*-space range of 0.6–3.5 Å. They were best-fitted using the two-shell model with eight structural parameters: each shell is described by 4 parameters–the coordination number (*N*), interatomic distance (*R*), mean-square relative displacement (MSRD) (σ^2^), also known as the Debye–Waller factor, and *C*_3_ cumulant which accounts for a deviation of the radial distribution function from the Gaussian shape. The backscattering amplitude and phase shift functions for Zn–O and Zn–Zn atom pairs were calculated using ab initio self-consistent real-space multiple-scattering (MS) FEFF8.50L code^63,64^ for wurtzite-type ZnO crystallographic structure. The scattering potential and partial phase shifts were calculated within the muffin-tin (MT) approximation^63,64^ for the clusters constructed based on the crystallographic structures of reference compounds with a radius of 8 Å, centered at the absorbing Zn atom. The inelastic losses of a photoelectron were accounted for using the complex exchange-correlation Hedin–Lundqvist potential.^65^

Note that the peaks in all FTs reported here are located at distances that are slightly shorter than their crystallographic values because the FTs were not corrected for the phase shift present in the EXAFS equation.

### 1.8 In Situ Fourier Transform Infrared Measurements

IR absorption measurements were performed using Nicolet iS50 FTIR Spectrometer (Thermo Fisher Scientific) in reflectance mode, with an MCT-A detector. The ALD chamber was equipped with a custom lid designed with a Si window that allowed IR beam deflection into the chamber using an array of Au-coated flat and parabolic optical mirrors. Polymer samples were prepared on Au-coated Si wafers to increase substrate reflectance. A measurement was taken every 30 s, averaging 16 repetitions (23.64 s, total) scanning from 650 to 4000 cm^–1^ in 4 cm^–1^ increments.

### 1.9 High-Resolution Scanning Transmission Electron Microscopy (HR-STEM)

HR-STEM imaging was done using Thermo Fisher Titan Cubed Themis G2 60–300 at 300 keV with a high-angle annular dark field (HAADF) detector. Camera length was set at 94 mm. Energy dispersive X-ray spectroscopy (EDX) was acquired with a Bruker Dual-X detector.

## Supporting Information Available

High-resolution STEM and FFT analysis of ZnO in SU-8 and PMCHO, STEM-EDX analysis, *in situ* FTIR of ZnO_*x*_ growth in PMMA, SU-8, and PMCHO, EXAFS detailed analysis, microgravimetric measurements of the growth, water absorption experiments (PDF)
