# Supplementary material for: The Development and Atomic Structure of Zinc Oxide Crystals Grown within Polymers from Vapor Phase Precursors
Source: ACS Nano. 2024 Jul 3;18(28):18393–404. doi: 10.1021/acsnano.4c02846 (PMC11256898; doi:10.1021/acsnano.4c02846)
Supplement: Supplementary file 1 — nn4c02846_si_001.pdf [file nn4c02846_si_001.pdf]

The development and atomic structure of zinc oxide crystals grown within polymers from vapor phase precursors

Supplementary Information

Inbal Weisbord,[1] Maya Barzilay,[1] Ruoke Cai,[1] Edmund Welter,[2] Alexei Kuzmin,[3] Andris Anspoks,[3] Tamar Segal-Peretz[1]

1 Department of Chemical Engineering, Technion – Israel Institute of Technology, Israel
2 Deutsches Elektronen-Synchrotron – A Research Centre of the Helmholtz Association, Notkestrasse 85, D-22607 Hamburg, Germany
3 Institute of Solid State Physics, University of Latvia, Kengaraga Street 8, LV-1063 Riga, Latvia

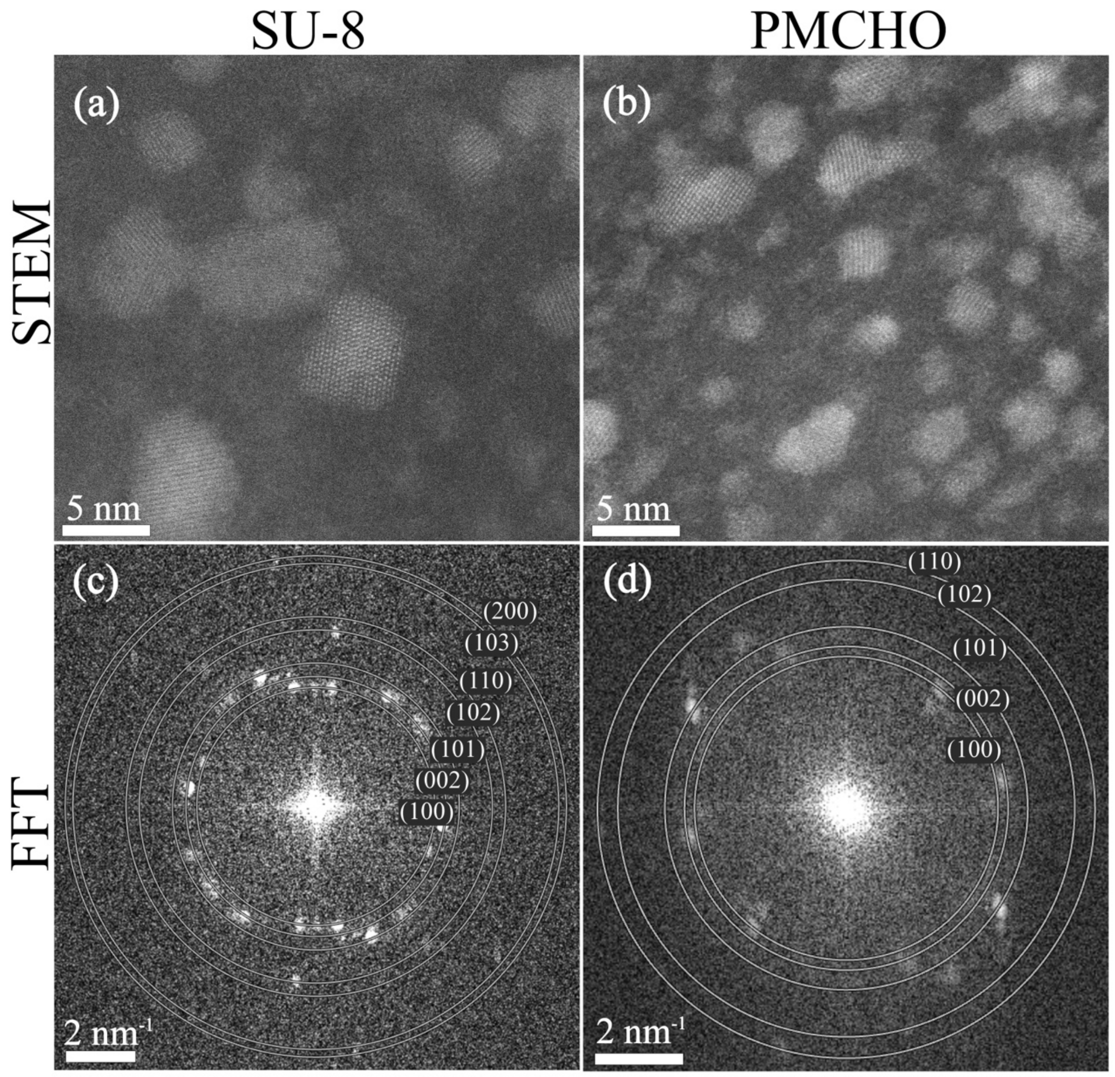

Figure S1. STEM FFT analysis: STEM images (a-b) and FFT patterns (c-d) for SU-8 (a, c) and PMCHO (b, d) after 5 SIS cycles. Rings drawn in the FFT patterns illustrate the periodicities found to match those of ZnO wurtzite-type d-spacings.

It is important to note that Figure b which shows atomic resolution imaging of PMCHO was obtained from a different sample type than that described in the methods section of the manuscript (SiN_x windows). PMCHO has proven to be much more sensitive to the electron beam than the other two polymers. For that reason, we were unable to obtain atomic resolution imaging of particles grown within the polymer on SiN_x. Instead, the polymer was spin-cast unto a water-soluble sacrificial layer on a Si wafer, which was then inserted into water. The PMCHO layer, which floated to the water surface, was then scooped onto a TEM grid, dried, and then inserted into the ALD chamber for the SIS process. The overall mass thickness of the sample (including substrate, polymer and ZnO) was lower than obtained on SiN_x windows, thus allowing atomic resolution imaging of ZnO_x crystals in PMCHO. Unfortunately, polymer film thickness was inconsistent when made in this manner, a fact that prompted our choice of SiN_x windows for HR-TEM study of ZnO SIS in these polymers.

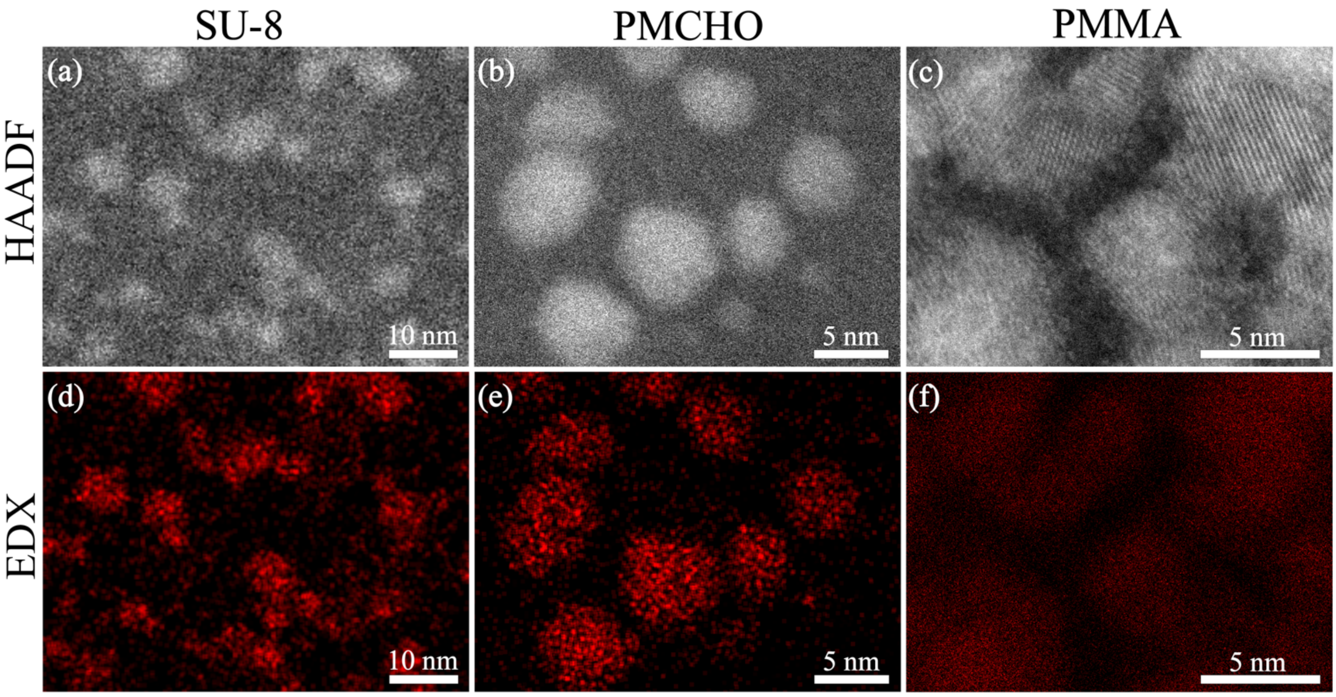

Figure S2. HAADF STEM (a-c) and Zn EDX mapping (d-f) of all three polymers: SU-8 (a,d), PMCHO (b,e) and PMMA (c,f).

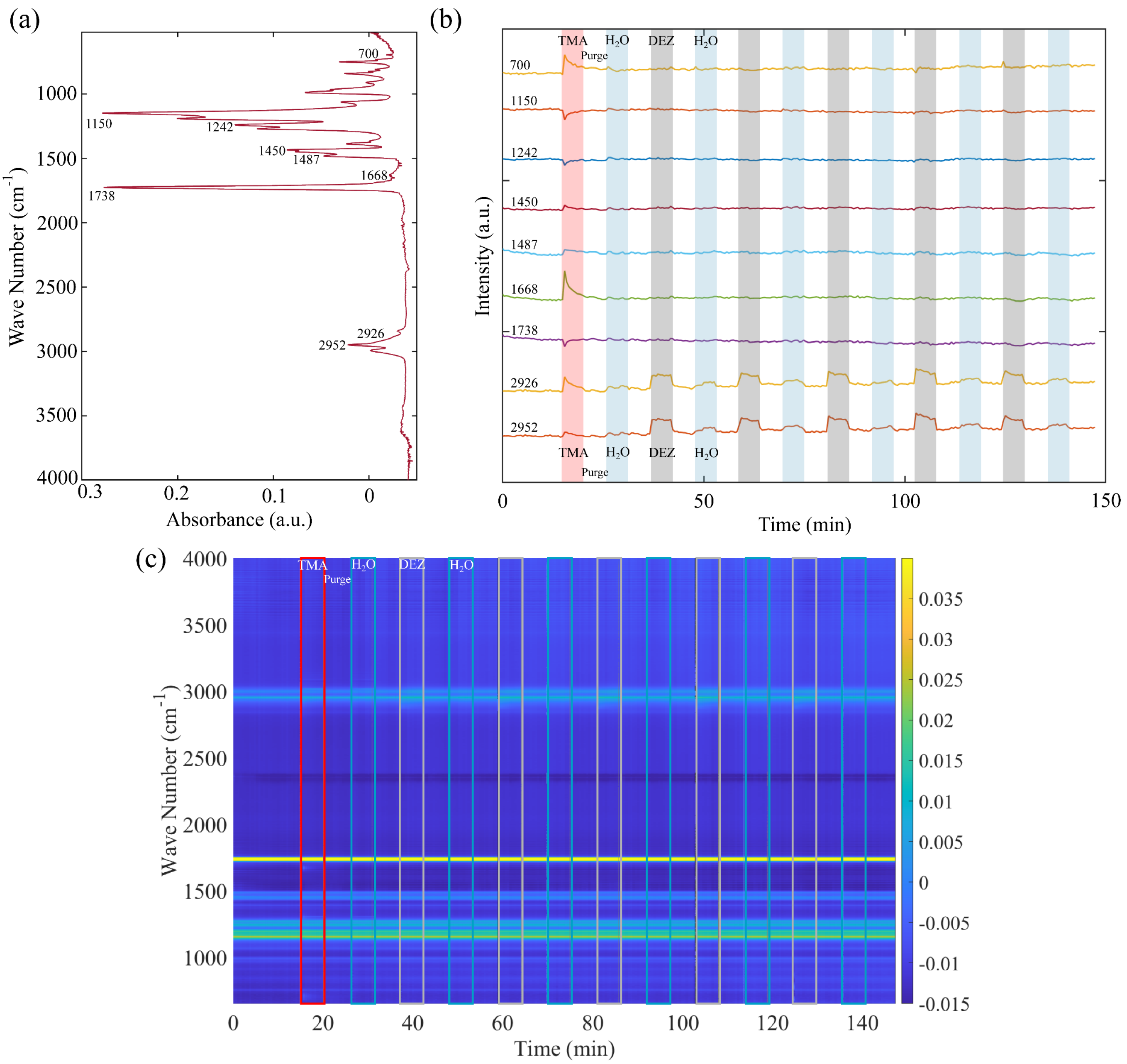

Figure S3. FTIR of PMMA: ex-situ (a) and in-situ (b, c) measurements. (a) shows a standard measurement of the pristine polymer, denoting relevant peaks. (b) and (c) show a measurement done during SIS, with one cycle of TMA/H_2O exposure, followed by 5 DEZ/H_2O exposures. TMA, DEZ and water are denoted by red, grey and teal, respectively. In (b) data is presented by following select peaks of interest as a function of time, while (c) shows the entire data in a height map form.

Table S1. PMMA IR signal peak assignment

**Wave number (cm^{-1})**	**Assignment**
700	TMA Al-C and AlO_x
1150, 1242	C-O-R stretch
1450, 1487	C-H bend
1668	C-O-Al stretch
1738	C=O stretch
2926, 2952	C-H stretch

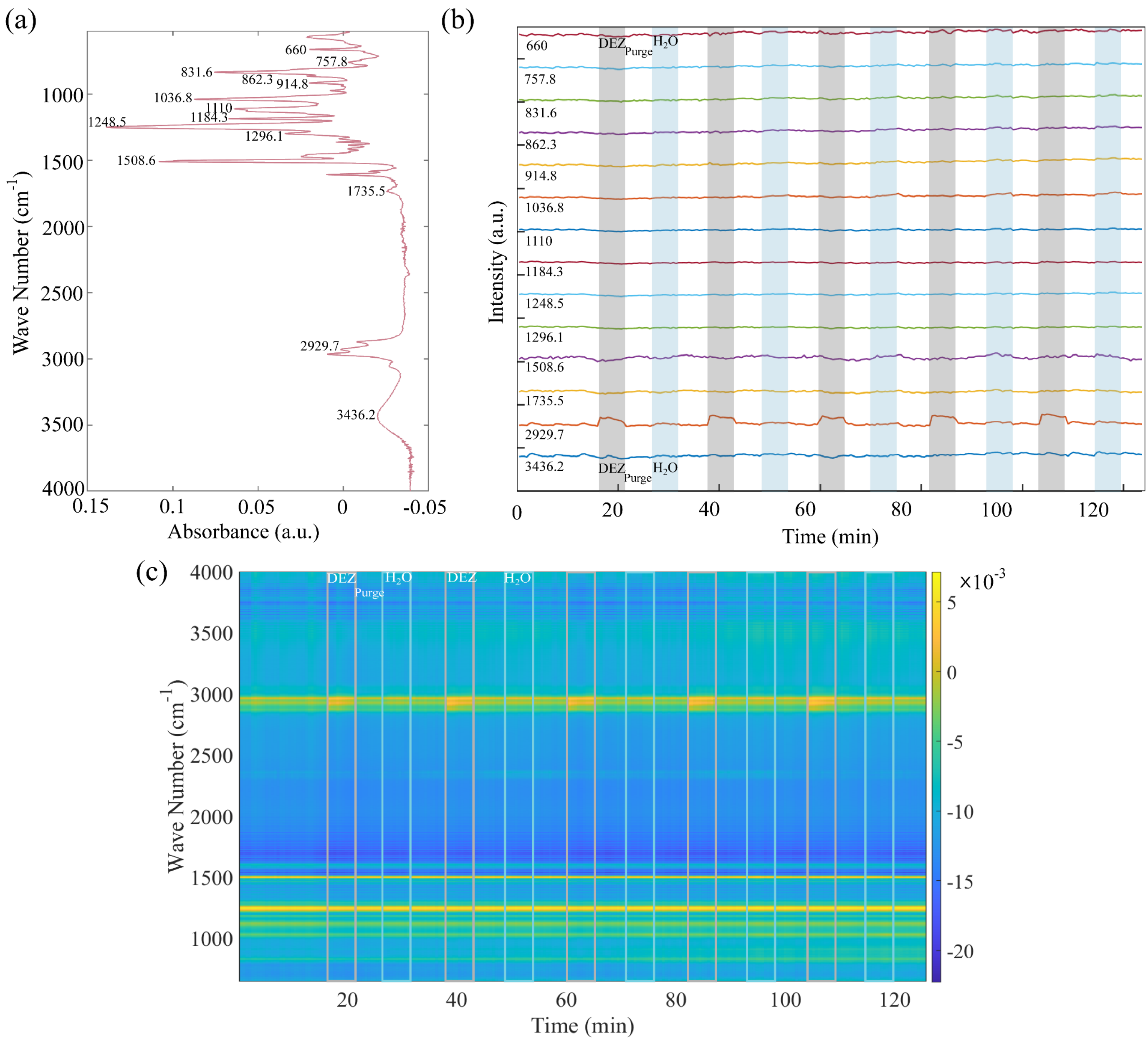

Figure S4. FTIR of SU-8: ex-situ (a) and in-situ (b, c) measurements. (a) shows a standard measurement of the pristine polymer, denoting relevant peaks. (b) and (c) show a measurement done during SIS, with 5 DEZ/H2O exposures. DEZ and water are denoted by grey and teal, respectively. In (b) data is presented by following select peaks of interest as a function of time, while (c) shows the entire data in a height map form.

Table S2. SU-8 IR signal peak assignment

Wave number (cm^{-1})	Assignment
660-862.3	ZnO_x
914.8	Epoxy ring C-O-C bend
1036.8, 1110, 1184.3	Ether
1508.6	Benzene
1735.5	Solvent carbonyl C=O stretch
2929.7	C-H stretch
3436.2	Hydroxy group (OH)

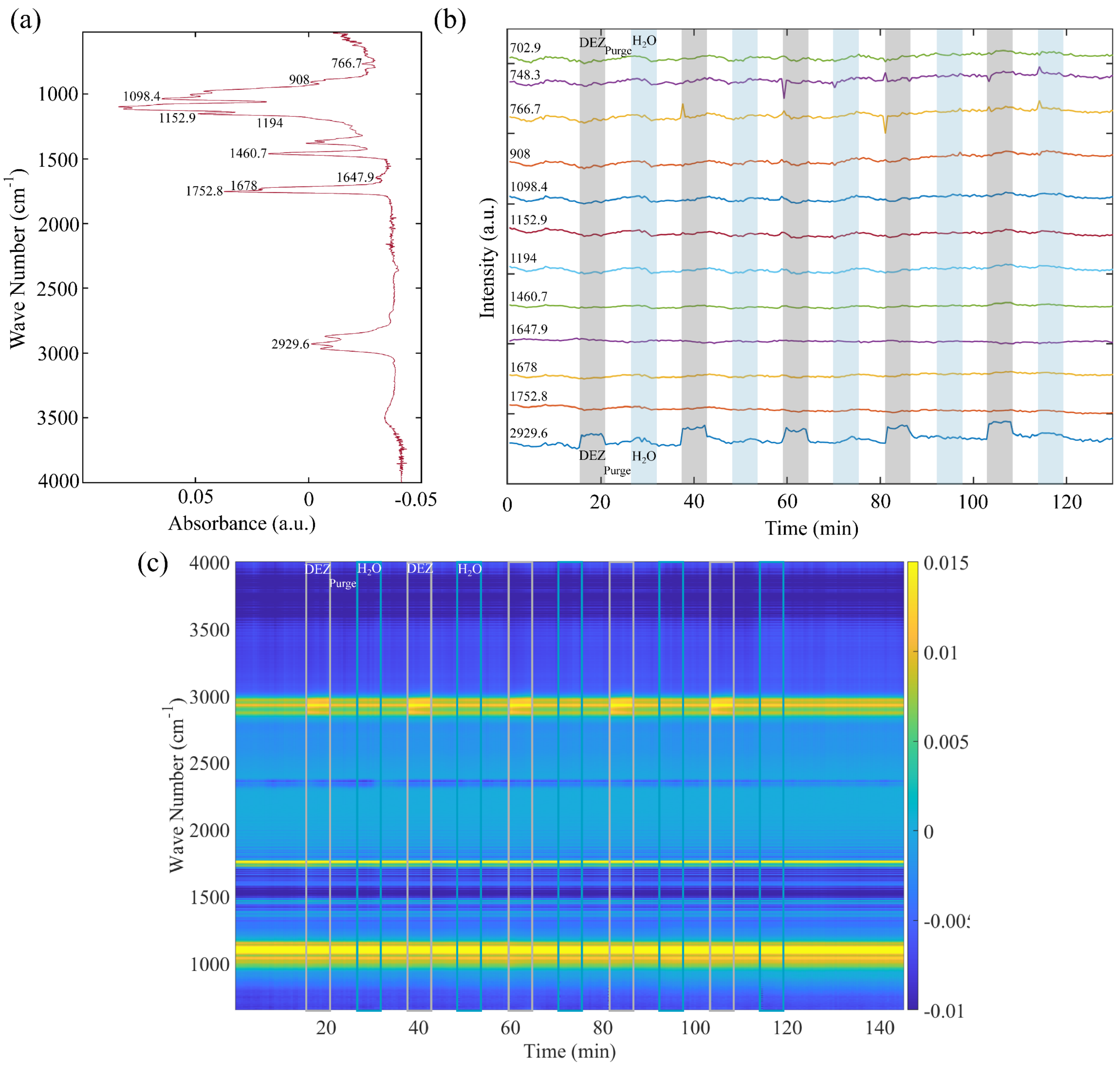

Figure S5. FTIR of PMCHO: ex-situ (a) and in-situ (b, c) measurements. (a) shows a standard measurement of the pristine polymer, denoting relevant peaks. (b) and (c) show a measurement done during SIS, with 5 DEZ/H_2O exposures. DEZ and water are denoted by grey and teal, respectively. In (b) data is presented by following select peaks of interest as a function of time, while (c) shows the entire data in a height map form.

Table S3. PMCHO IR signal peak assignment

Wave number (cm^{-1})	Assignment
702.9-908	ZnO_x
1152.9, 1194	C-OH stretch
1647.9	C-O-Zn
1678, 1752.8	C=O stretch
2929.6, 3020	C-H stretch

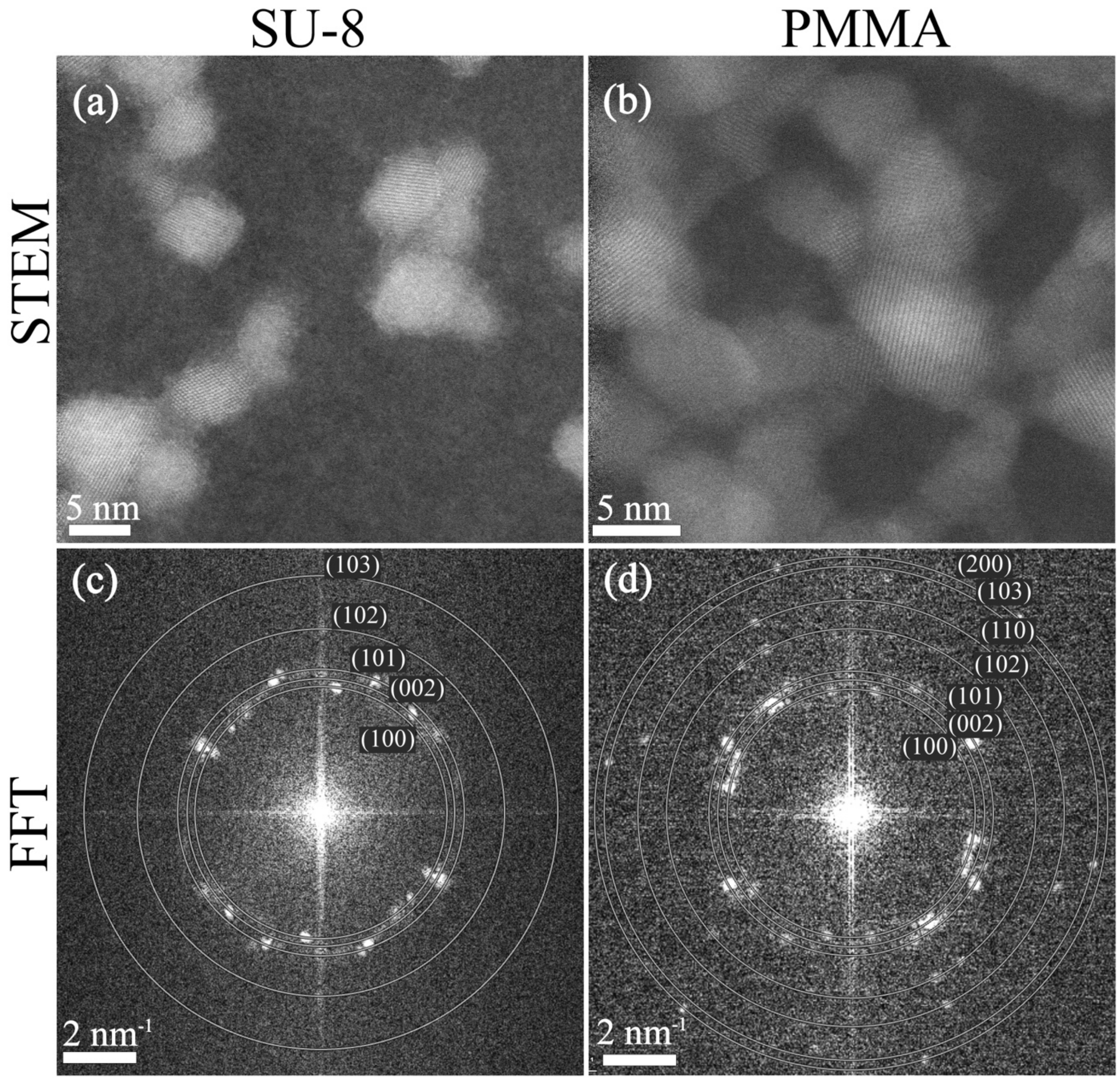

Figure S6. Thermal treatment STEM FFT Analysis: STEM images (a-b) and FFT patterns (c-d) for SU-8 (a, c) and PMMA (b, d). Rings drawn in the FFT patterns illustrate the periodicities found to match those of ZnO wurtzite-type d-spacings.

Table S4. Structural parameters for the first and second coordination shells of Zn obtained from the fitting of the Zn K-edge EXAFS spectra. Here N is a coordination number (±0.4), R is an average interatomic distance (±0.02 Å), MSRD is mean-square relative displacement (σ^2, ±0.002 Å^2), also known as the Debye-Waller factor, and C_3 is a third cumulant (±0.0005 Å^3) which accounts for a deviation of the radial distribution function from the Gaussian shape. The table compares data acquired after 5 SIS cycles, and after 5 cycles and 600°C thermal treatment.

		**SU8**		**PMCHO**		**PMMA**	
	Number of cycles	5	5+oven	5	5+oven	5	5+oven
Zn-O	N	3.5	3.4	3.6	3.8	3.7	3.6
	R	2.01	2.00	2.01	1.98	2.02	1.99
	MSRD	0.005	0.003	0.007	0.006	0.007	0.005
	C3	0.001	0.001	0.001	0.001	0.002	0.002
Zn-Zn	N	**12.0**	**12.0**	**12.0**	**12.0**	12.0	12.0
	R	3.44	3.31	3.46	3.28	3.44	3.37
	MSRD	**0.039**	**0.017**	**0.039**	**0.013**	0.034	0.023
	C3	0.006	0.002	0.007	0.001	0.006	0.003

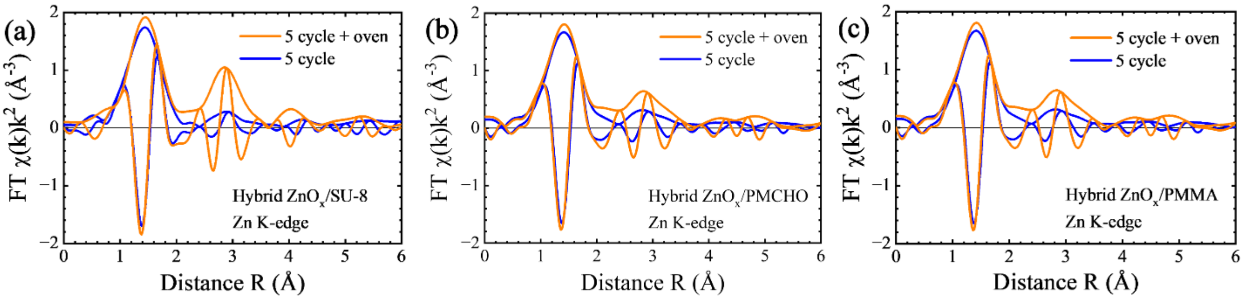

Figure S7. Comparison between hybrid polymer-metal oxide and inorganic films. Modulus and imaginary parts of Fourier transforms (FTs) of the Zn K-edge EXAFS spectra for Hybrid ZnO_x/SU8 (a), ZnO_x/PMCHO (b) and ZnO_x/PMMA (c) polymers after 5 cycles (blue) and burning at 600°C in air (orange).

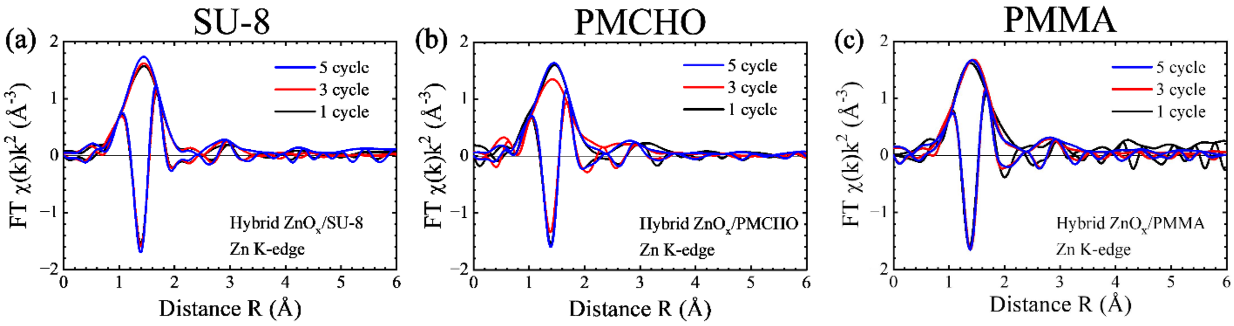

Figure S8. Growth evolution with cycles: modulus and imaginary parts of Fourier transforms (FTs) of the Zn K-edge EXAFS spectra for hybrid ZnO_x/SU8 (a), ZnO_x/PMCHO (b) and ZnO_x/PMMA (c) after 1 (black), 3 (red), and 5 (blue) cycles.

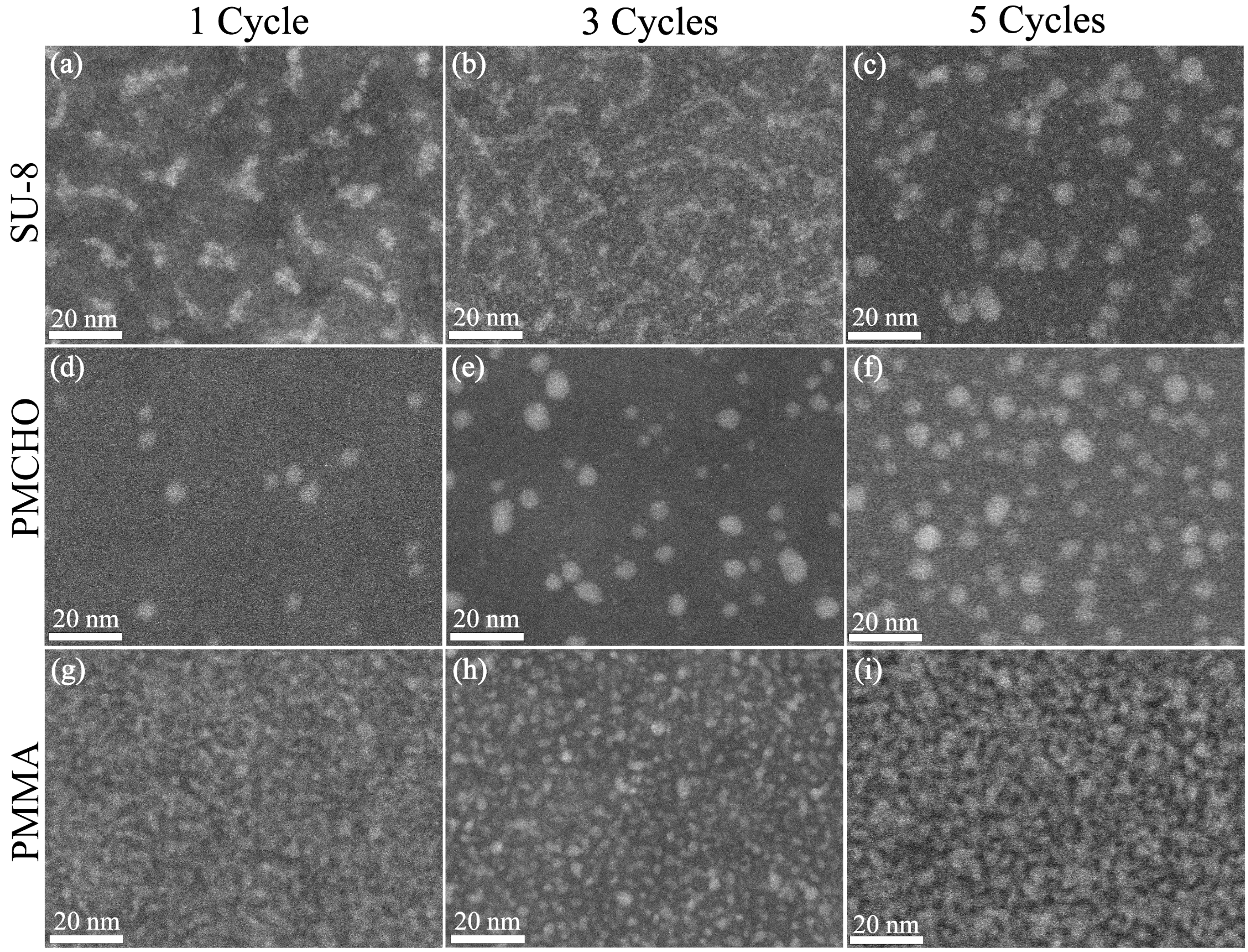

Figure S9. Growth evolution with cycles in SU-8 (a-c), PMCHO (d-f) and PMMA (g-i): STEM images of hybrid ZnO_x/polymer after 1 (a, d, g), 3 (b, e, h), or 5 (c, f, i) cycles.

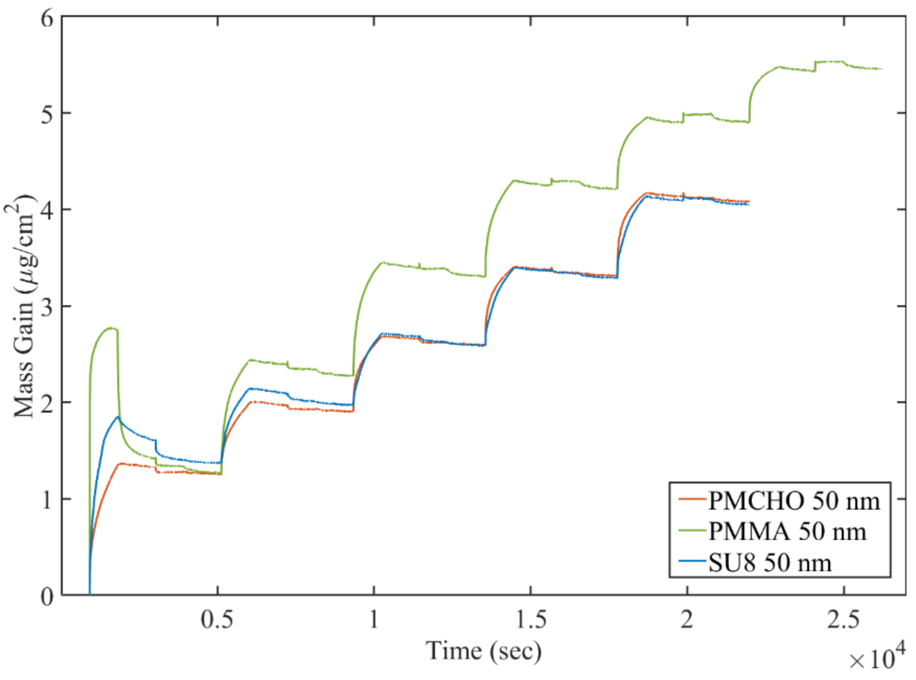

Figure S10. Representative QCM measurements of growth in different polymers. Experiments were done at 120°C. Each cycle was comprised of 900 s exposure to the organometallic precursor, followed by 1200 s N_2 purge and then 900 s exposure to water, followed by 1200 s N_2 purge. Exposures were done in static mode, during which the chamber is completely closed and there is no N_2 flow.

The first cycle in the PMMA experiment (green plot) was done using trimethyl aluminum (TMA) as the organometallic precursor. To gain statistically significant data, each experiment was repeated a few times, and the averaged results are shown in the main text.

Water Absorption Experiments

In order to evaluate the water intake (or hygroscopy) of the polymers, we conducted QCM water exposure experiments (**Figure 8**). After preparation (described in the methods section), which included sample drying in a desiccator for at least 2 hours, each sample was kept in a N_2 glove box. Prior to each experiment, the sample was placed in the ALD at 120°C for at least 4 hr at 50 sccm N_2 flow, to eliminate water residues from the atmosphere. Stabilization ended and the experiment began only when frequency variations were **lower than 1 Hz during a 5 hr period**. Experiments consisted of three water/purge half cycles. The pressure profile of this experiment, measured using capacitance manometer in a PMMA experiment together with details on the flow at each stage and the valve position, can be found below in Figure S11e. In each half cycle the sample was exposed to water vapors, during which time the chamber was sealed in a completely static mode for 300 s. Chamber valves were then opened, and the chamber was purged for 300 s with 20 sccm N_2 flow. Prior to these three half cycles, a similar half cycle was done once without opening the water valve, as a control

experiment meant to evaluate the effect of N_2 pressure and valve opening and closing on the mass measurement (for example, at 5400 sec, N_2 flow is decreased from 50 sccm, which is the instrument flow rate during the drying stage, to 5 sccm – the flow rate preceding each pulse). A word of caution- these measurements are close to the sensitivity limit of the in-situ QCM and should be performed on a stable polymer film and a stable QCM to obtain repeatable results.

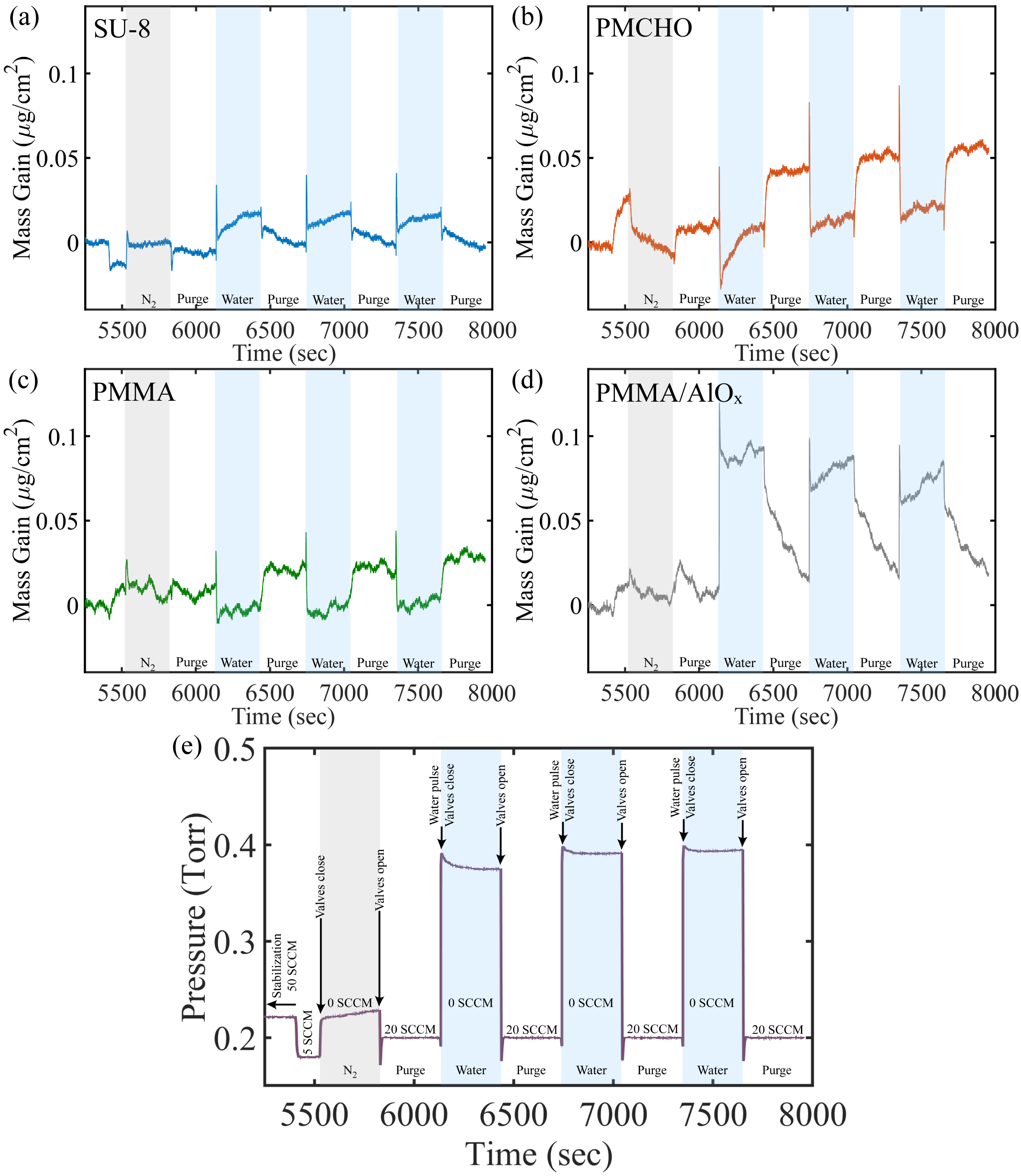

Figure S11. QCM water uptake evaluation raw data for SU-8 (a), PMCHO (b), PMMA (c) and the PMMA/AlO_x hybrid (d), represented on separate axes. Experiments were done at 120°C. Each cycle was comprised of 300 s

exposure to water, followed by 300 s N_2 purge. Before the first exposure to water, a single cycle was performed without opening the water valve, to help eliminate N_2 and chamber valve procedure artifacts (gray area). Exposures were done in static mode, during which the chamber is completely closed and there is no N_2 flow. (e) A typical pressure profile, valve position, and flow through the chamber during the water uptake experiments.
